# High‐molecular‐weight oligomer tau (HMWoTau) species are dramatically increased in Braak‐stage dependent manner in the frontal lobe of human brains, demonstrated by a novel oligomer Tau ELISA with a mouse monoclonal antibody (APNmAb005)

**DOI:** 10.1096/fj.202401704R

**Published:** 2024-11-20

**Authors:** Hiroaki Fukumoto, Tzu‐Huei Kao, Chin‐Yin Tai, Ming‐Kuei Jang, Masaomi Miyamoto

**Affiliations:** ^1^ Department of Preclinical Research Division APRINOIA Therapeutics Inc. Tokyo Japan; ^2^ APRINOIA Therapeutics Inc. Taipei China

**Keywords:** Alzheimer, APNmAb005, Braak‐stage, conformation‐specific, ELISA, globular oligomer, HMWoTau, p181Tau, total Tau

## Abstract

Disease‐specific oligomers Tau assay system is anticipated in Alzheimer disease (AD) to elucidate their etiological roles. We developed a highly sensitive and selective ELISA for high‐molecular‐weight oligomer tau (HMWoTau) with LLOQ of 0.3 pg/well for the first time, using a novel mouse monoclonal antibody APNmAb005. The target molecule was identified as HMWoTau with circa 2000 kD as a minimum size and the more oligomerized species (>5000 kD), in combination analysis with Size‐Exclusion‐Chromatography and Sucrose‐Density‐Gradient‐Centrifugation for both recombinant human (rh) Tau‐derived aggregates and AD brain‐lysates in PBS(−). HMWoTau was labeled by Thioflavin S and visualized as a homogeneous globular particle (about 30 nm in diameter) by two different technologies of atomic force microscopy and dSTORM‐Nanoimager. Specific quantitation was also confirmed by immune‐absorption, rhHMWoTau‐spiked, and cross‐reactivity studies. APNmAb005 failed to detect the HMWoTau signal by treatment with DTT/SDS under no influence on the pan‐tau antibody, indicating its conformation‐specific recognition. APNmAb005‐ELISA showed AD‐specific and statistically significant ELISA signals from 1 ng brain lysate protein/well. Analysis of the frontal neocortex (*N* = 40, Braak stage I–VI) by ELISA revealed the detection‐limit levels of HMWoTau species at stage I–III, and drastic and statistically significant increases at stage V/VI (AD). By contrast, total Tau and p181 Tau showed 1/4–1/5 levels of AD even at Stage I, while both tau species also showed a statistically significant increase in AD. In sum, our novel APNmAb005‐ELISA clarified the disease‐specific increase in HMWoTau species and will be useful for not only further etiological elucidation but also the potential diagnostics in AD and relevant tauopathy.

## Graphical abstract

HMWoTau species‐specific APNmAb005‐ELISA was developed with a detection limit of 0.3 pg rhHMWoTau/well, enabling AD‐specific signal detection from 1 ng brain lysate protein/well. APNmAb005's target was identified as HMWoTau (approximately 2000 kD, about 30 nm in diameter) and another more oligomerized tau species (roughly estimated as 7‐fold length). Human frontal lobe (Braak Stage I–VI) showed statistically significant increases in HMWoTau species at Braak stage V/VI of AD under the detection‐limit levels at stage I–III, indicating HMWoTau species' disease‐specific generation.

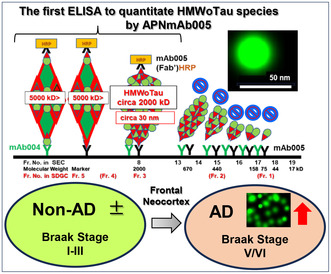

**Abbreviations**
AD: Alzheimer's disease
AFM: atomic force microscopy
CBD: Corticobasal degeneration
dSTORM: direct Stochastic Optical Reconstruction Microscopy
DTT: dithiothreitol
ELISA: enzyme‐linked immunosorbent assay
FRET: fluorescence resonance energy transfer
HAMA: Human‐Anti‐Mouse‐Antibody
HMWoTau: high‐molecular‐weight oligomer Tau
LLOQ: Lowest Limit of Quantification
LMWoTau: low‐molecular‐weight oligomer Tau
mAb: monoclonal antibody
MMWoTau: middle‐molecular‐weight oligomer Tau
mTau: monomer tau
oTau: oligomer tau
PBS(−): phosphate buffered saline without Ca^++^/Mg^++^
PET: positron emission tomography
PSP: progressive supranuclear palsy
rh: recombinant human
SDGC: Sucrose‐Density‐Gradient‐Centrifugation
SDS: sodium dodecyl sulfate
SDS‐PAGE: sodium dodecyl sulfate‐polyacrylamide gel electrophoresis
SEC: Size‐Exclusion‐Chromatography
thioflavin S: Thio‐S

## 1 INTRODUCTION

Alzheimer's disease (AD) has been pathologically confirmed by the presence of Aβ plaque and neurofibrillary tangles, and both pathologies have gained attraction from viewpoints of AD etiology as well as target molecules aiming for the development of disease‐modifying drugs.^1^, ^2^, ^3^ In fact, in the case of Aβ‐focused drug discovery, Aβ‐protofibril selective monoclonal antibody Lecanemab and pathologically specific antibody Donanemab are successful cases approved as a disease‐modifying drug based on their clinical trial results.^4^, ^5^

Recently discovered Tau PET recapitulated the Braak‐stage dependent tau‐pathology, in general.^6^, ^7^, ^8^ Tau pathology has been considered to be more correlated with AD dementia scores/neuronal loss than Aβ pathology and this concept was further ensured in live subjects using tau PET and Aβ PET.^9^, ^10^, ^11^ Because Aβ pathology has been reported to appear before the onset of AD,^12^ and also to precede to tau pathology as well as neuronal loss in AD,^3^ the modulation of Aβ pathology needs to be started in the earlier stage of AD or before the onset of AD, ideally. On the other hand, modulating tau pathology of AD seems to be more controllable than Aβ pathology because tau pathology appears later than Aβ pathology and continues to expand during devastating disease progression with neuronal loss and cognitive impairment, once diagnosed as AD.^3^

“Neurofibrillary tangles” are the most advanced forms of aggregates derived from monomer tau protein in tau pathology. Recent studies shed light on intermediate oligomer species (diameter < 50 nm)^13^ rather than “Neurofibrillary Tangles” because of their roles as a seed of tau propagation,^14^, ^15^, ^16^, ^17^, ^18^, ^19^ as well as their neurotoxic effects in tau‐associated neurodegeneration.^15^, ^17^, ^20^, ^21^, ^22^ However, we do not know clearly about roles of each tau oligomer species, such as dimer and trimer (<150 kD) of low ‐molecular‐weight oligomer tau (LMWoTau), middle‐molecular‐weight oligomer tau (MMWoTau) (<670 kD), or high‐molecular‐weight oligomer tau (HMWoTau) (circa 2000 kD). It still needs to be clarified which tau species in pathologically native conditions could be a real culprit of tau propagation and/or tau‐associated neuronal death in AD, in spite of accumulating reports.^23^ In fact, we still see some debate on responsible tau molecular species on tau propagation.^24^, ^25^

In published articles,^23^ LMWoTau has been considered as a candidate for toxic tau species. Recent discovery of synaptic protein Bassoon elucidated its association with HMWoTau, but not with LMWoTau, in brain lysates from AD and progressive supranuclear palsy (PSP). The knock‐down of Bassoon in animal models not only inhibited tau propagation but also protected neuronal loss,^17^ implying HMWoTau could be one candidate molecule to exert tau propagation as well as toxic effects in animals and human. Therefore, we need to continue to elucidate responsible molecular species of tau and their roles in a more elaborate manner using tau oligomer‐specific assay system.

One of the reasons for difficulties in identifying causative tau protein species seems to lie in the fragile/unstable nature of oligomer/aggregate molecules, which should be taken into consideration during assay procedures. To estimate molecular weights and protein levels for target molecules, we have a handful of assays including the most popularly used western blot (WB) analysis, which has been also applied to many tau oligomer/fibril protein studies in the past. However, WB analysis employs harsh sample treatments using protein‐complex‐disrupting reagents such as sodium dodecyl sulfate (SDS) and reducing reagents such as dithiothreitol (DTT) or 2‐mercaptoethanol, together with heat‐denature, leading to ablation of S–S bond and to generation of disassembled molecular species. The pre‐treatments of samples finally result in different conformations/forms from those originally existing in physiological/pathological conditions. Thus, WB analysis is not suitable to characterize harsh‐treatment‐sensitive proteins such as oligomers and protofibrils.

By contrast, ELISA, dot blot assay, and fluorescence resonance energy transfer (FRET) assay employing specific antibodies enable us to detect fragile oligomer complexes under the conditions of physiological buffers such as phosphate‐buffered saline without detergent/S–S bond brakers, while it lacks the information of molecular size, unless conducting analysis of MW size using such as Size‐Exclusion‐Chromatography (SEC) and/or Sucrose‐Density‐Gradient‐Centrifugation (SDGC), together with their calibrated standard molecular weight markers.

In order to detect oligomer tau species, several specific antibodies have been actually generated in the past. For example, the following antibodies could be listed: MC1,^26^ TOC1,^27^ T22,^28^ TOMA,^29^ and M204.^30^ However, the target oligomer species in native forms do not seem to be clearly identified nor documented well in most cases.

We have successfully discovered a disease‐specific mouse monoclonal antibody APNmAb005 (hereafter, called “mAb005”) using oligomerized rhTau (2N4R) as antigens (submitted). mAb005 was able to show its preferential binding to HMWoTau species in dot blot analysis. mAb005 also immunologically detected tau‐pathologies in AD, progressive supranuclear palsy (PSP), Pick disease, and corticobasal degeneration (CBD) brains. In preclinical study, mAb005 has exerted its neuroprotective effects in rTg4510, being the most severe animal model in tauopathy.^31^, ^32^, ^33^ Now, its humanized monoclonal antibody of mAb005 has moved into the clinical trial aiming for AD treatment (see NCT05344989, entitled “A First‐in‐Human Study to Assess Single Doses of APNmAb005 in Healthy Participants” in ClinicalTrial.gov).

Throughout this study, we only focused on detergent‐free PBS(−)/Tris buffer‐extractable molecules, because oligomer/aggregates are sensitive to strong detergent and S–S bond breaker as mentioned above. Furthermore, cell culture studies revealed that oligomer tau is secreted out from cells^34^ and such a detergent free‐PBS(−) extractable oligomer species seems to play more pivotal roles in tau propagation/neuronal toxicities, rather than insoluble fractions of tau species.

As far as we know, this is the first report of the establishment of HMWoTau species‐ specific ELISA consisting of mAb005. We also successfully generated mAb005‐ELISA's target protein of rhHMWoTau as a calibration standard. Furthermore, we were able to quantitate and detect PBS(−)‐extractable HMWoTau species in AD brain lysates from the frontal lobe, where tau‐propagation is considered to reach finally, as visualized by a new PET tracer Florzolotau (18F).^35^, ^36^, ^37^, ^38^

## 2 MATERIALS AND METHODS

### 2.1 Generation of recombinant human tau (rhTau)

The isoform of human tau (2N4R) among six tau isoforms^39^, ^40^ was expressed in *E. coli* (One Shot BL21 Star DE3, Thermo Scientific) using the pRK172‐htau40 plasmid, because mAb005 monoclonal antibody was generated against the oligomerized 2N4R species as antigen source (submitted). The bacteria were lysed in 20 mM Tris‐buffer (pH 9.0) by sonication. The lysate was centrifuged at 15 000 *g* for 20 min. The supernatant was filtered with 0.45 μm and subjected to anion exchange chromatography (Cytiva HiTrap Q HP column) with gradient elution buffer (0–250 mM NaCl) in ÄKTA fast protein liquid chromatography (FPLC) system, and then the eluted materials were further subjected to Size‐Exclusion‐Chromatography (SEC) (Cytiva HiLoad 16/600 Superdex 200 column) with elution buffer of 20 mM Tris‐buffer (pH 7.4). Tau monomer was obtained and protein concentration was determined at OD280 nm using NanoDrop Lite (Thermo Scientific) with an extinction coefficient of 7700 M^−1^ cm^−1^ as reported^41^ and the aliquots (>10 mg/mL) were made in storage buffer (25 mM HEPES, pH 7.2, 0.1 mM EDTA, 0.5 mM DTT, 100 mM NaCl) and stored at −80°C until use for monomerization as well as oligomerization studies.

### 2.2 Size‐exclusion‐chromatography (SEC)

Samples were re‐centrifuged at 10 000 g for 15 min when frozen samples were thawed from the freezer to get rid of the potential insoluble for column protection. Then, the supernatant adjusted with protein concentration was loaded into Superose 6 Increase 10/300 GL column (Cytiva, Cat. No. 29‐0915‐96) (fractionation range from 5 to 5000 kD for globular proteins), mounted on ÄKTA Purifier (GE Healthcare) and fractionated at a flowrate of 0.5 mL/min at a volume of 0.5 mL/fraction or of 1 mL/fraction as specified in figures using PBS(−) as elution buffer in principle, except generation of rhTau. The protein level was monitored by OD280 nm and/or each fractionated protein level was determined using NanoDrop Light (Thermofisher) with BSA as a standard. Blue Dextran 2000 (GE Healthcare, Cat No. 17‐0360‐01), AdavnceBio SEC 300A Protein standard (Agilent Technology, Cat No. 5190‐9417), and/or gel filtration calibration kit HMW (Cytiva, Cat. No. 28403842) were used as molecular weight standards for SEC.

### 2.3 Sucrose density gradient centrifugation (SDGC)

SDGC was conducted in a similar way as published.^42^, ^43^ Briefly, sucrose (Fujifilm Wako, Cat. No. 190‐0001) was dissolved in Milli‐Q water to obtain 10%, 20%, 30%, 40%, and 50% (w/v) sucrose solution. Each 1 mL of sucrose solution, except 0.8 mL of 10% sucrose solution was layered into a 5 mL centrifuge tube (Beckman, Cat No. 326819), starting with 50%, and then followed by 40%, 30%, and 20% sucrose, until 10% sucrose, to prepare discontinuous gradient of sucrose solution on ice. Each equal amount of thawed sample of 20 μg/200 μL diluted with PBS(−) on monomer rhTau, fibril tau mixtures, and rhHMWoTau, or 400 μg/200 μL diluted with PBS(−) on human brain lysate from NAD/AD was carefully loaded onto the top of 10% sucrose solution and centrifuged using Optima MAX‐XP Ultracentrifuge (Beckman Coulter) at 200 000 g in a swing rotor (Beckman, MLS‐50) for a total time of 2 h at 4°C. Then, each layer of 10%, 20%, 30%, 40%, and 50% sucrose fractions was carefully collected from top to bottom by 1 mL/fraction for recombinant tau species, named as Fr. X (*X* = 1–5, corresponding to each sucrose fraction of 10%–50%) or by 500 μL/fraction for human brain lysates to get more precise separation, and named as Fr. X‐1 (top side), Fr. X‐2 (bottom side) (*X* = 1–5, corresponding to each sucrose fraction of 10%–50%). Each pellet was dissolved in 500 μL Milli‐Q water and collected after 20 times pipetting (Fr. 6‐1). Then, another 500 μL of Milli‐Q water/0.01% Tween 20 was added into the tube. The remained pellet was sonicated 1 s for 5 times by Ultrasonic Homogenizer (Smart NR‐50, MICROTEC Co. Ltd., Japan) and collected as Fr. 6‐2. The sonication was ensured not to disrupt rhHMWoTau by ELISA (data not shown). For rhTau species, Fr. 6‐1 and Fr. 6‐2 was mixed as Fr. 6.

### 2.4 Monomerization of tau oligomers

Recombinant human Tau (2N4R), containing preformed oligomers probably due to stock at a high concentration of above 10 mg/mL, was monomerized as follows. The PBS(−) mixtures (500 μL) of rhTau (500 μg/mL), 5 mM DTT (Fujifilm Wako, Cat. No. 047‐08973), and 1 mM EDTA (Fujifilm Wako, Cat. No. 345‐01865) in polypropylene tube were heated for 90 min at 60°C. This aliquot of mixture was stored at −80°C until use. The aliquot was confirmed as monomer tau by SEC (data not shown) and Sucrose Density Gradient Centrifugation (SDGC) in combination with total Tau ELISA (see the result section).

### 2.5 Generation of tau fibril mixtures

Total mixtures (500 μL) consisting of rhTau (diluted to 4 mg/mL with PBS(−)), heparin sulfate (400 μg/mL PBS(−), Sigma, Cat. No. H3393‐100KU) and (±)DTT (2 mM in PBS(−), Fujifilm‐Wako Cat. No. 043‐08793) were incubated in polypropylene tube at 37°C for 14 days, referring to the report,^44^ except addition of 1 mM of freshly prepared DTT every 24 h. The aliquot of incubated mixture was made and stored at −80°C until analysis by SDGC and ELISA.

### 2.6 Generation and purification of recombinant human HMWoTau (rhHMWoTau)

We incubated the mixture of stocked rhTau (diluted to above 4 mg/mL with PBS(−)) and heparin sulfate (above 400 μg/mL PBS(−)), being a plateau level (7.4%–10% of total protein concentration) at 37°C for 2–3 day. The supernatant of 500 μL was obtained after centrifugation of the reaction mixture at 10 000 *g* for 15 min, then subjected to SEC to obtain a single peak with MW of circa 2000 kD at Fr. 8. The aliquots of Fr. 8 were made and stored at −80°C until use to avoid freeze–thaw at a concentration of 100 μg/mL in PBS(−) for SDGC study, or 200 ng/mL in PBS(−) containing 0.1% BSA (protease free/fatty acid free/essentially globulin free, Sigma, Cat. No. A7030‐50G) for ELISA standard. Purified HMWoTau protein concentration was determined at OD280 nm using an extinction coefficient of 7700 M^−1^ cm^−1^ as reported.^41^

### 2.7 Monoclonal antibodies and conjugation with Horse‐radish‐peroxidase (HRP)

Respective 100 μg content of mouse monoclonal antibodies were conjugated with HRP using SureLink HRP conjugation kit (Thermo Scientific, Cat No. 84‐00‐01) according to the manufacturer's instruction for mAb005 (AD‐specific antibody, mouse monoclonal antibody, IgG1κ, APRINOIA) (submitted), mAb004 (in‐house pan‐tau antibody, N‐terminal, mouse monoclonal antibody, IgG2bκ, APRINOIA), and HT7 (mouse monoclonal antibody, IgG1κ, Thermo Scientific, Cat. No. MN1000), after buffer exchange of antibody solution to reaction buffer using Amicon ultra‐0.5 centrifugal filter unit (Merck, Cat. No. UFC501008). mAb005 (Fab’)HRP were prepared at Fujifilm Wako (Tokyo, Japan), using Fab’ fragment's SH functional moiety for cross‐linking with HRP‐maleimide, to avoid inactivation of antigen‐recognition sites through amine or carboxy‐terminals by cross‐linking. All of the aliquots were stored in a refrigerator until use.

### 2.8 Sample treatment for tau ELISAs

Samples were diluted to ng levels of total lysate protein/well for human brain sample or pg levels of Tau protein/well for rhTau‐derived HMWoTau, total Tau, and p181Tau, respectively, as specified elsewhere. To see the effects of DTT/SDS and heat‐denature on tau antibody binding, samples were pretreated with or without heat‐denature at 95°C for 5 min in the presence or absence of 2.5 mM DTT and 2%SDS. In advance of ELISA, samples containing DTT/SDS or sucrose were diluted 400 times and above with specified buffer for ELISAs under confirmation of their no interference in ELISA (data not shown).

### 2.9 Two‐site sandwich ELISA

To quantitate total Tau and p181Tau, we used commercially available human total Tau ELISA kit (Cat. No. 27811) and human phospho tau (181P) ELISA kit (Cat. No. 27812) (Immuno‐biological laboratories (IBL), Co., Ltd., Japan) according to the manufacturer's instructions,^45^ together with some modifications as specified below. Namely, we replaced the capture antibody with mAb004 (in house pan‐tau antibody, IgG2bκ, epitope; N‐terminal); the ELISAs consist of capture‐detector antibodies of Ab004‐IBLpanTau mAb (anti‐Tau441 clone E21A5A1)Fab'HRP (designated as IBLpanTau(Fab’)HRP) and mAb004‐IBLp181Tau (anti‐hTau p181 Rk27A6 rat monoclonal antibody)Fab'HRP (designated as IBLp181Tau(Fab’)HRP), respectively.

To quantitate HMWoTau, we used the following ELISAs consisting of capture‐detector antibodies; mAb005‐mAb005(Fab’)HRP, mAb004‐mAb005(Fab’)HRP, HT7‐mAb005 (Fab’)HRP, or mAb005‐IBLpanTau(Fab’)HRP. As a control commercially available oligomer Tau antibody, T22 (Merck Millipore, Cat. No. ABN454, Lot. 3845658) was used as a capture antibody. The assay buffers and assay procedures were similar to those described in the article on Aβ ELISA^46^ with some modifications.

Firstly, ELISA plate (Fluotrac 96‐well black plate, high‐binding, Greiner, Cat. No. 655077) or (Maxisorp 96‐well immune standard modules, black plate, Nunc, Cat. No. 475515) was coated with monoclonal antibodies of mAb005 (1–5 μg/mL), mAb004 (1–5 μg/mL), or HT7 (1 μg/mL) at a volume of 100 μL/well in coating buffer A (carbonate buffer, pH 9.6, 100 mM). After washing plates with PBS(−) using plate washer (AMW‐3 Auto Mini Washer, Bio Tec, Japan), each well was blocked with 120 μL of 1% (W/V) BlockAce (KAC Co. Ltd., Cat. No. UKB40, Japan) solution in PBS(−), pH 7.4. Then, the plate was stored at 4°C until use.

After washing the plate with PBS(−), 50 μL of sample or ELISA standard protein in PBS(−)/0.1% protease‐free BSA (Sigma, Cat. No. A7030‐50G) was added into each well, preloaded with 50 μL of sample diluent buffer B. The diluent buffer B consists of CHAPS, EDTA, protease‐free BSA, and high sault NaCl in PBS(−) pH 7.4 as reported,^46^ and additionally containing HAMA Blocking reagent (Cosmo Bio, Cat. No. 85R‐1001P) for human samples to eliminate non‐specific binding derived from HAMA (human anti‐mouse antibody). The plate was incubated overnight at 4°C.

For HMWoTau detection, after washing with PBS(−), mAb005(Fab’)HRP (5000× dilution) was added into each well at a volume of 100 μL in Intercept (PBS) Blocking Buffer (LiCor, Cat. No. 927‐70001) containing HAMA Blocking reagent. The plate was incubated at cold room overnight. For total Tau and p181Tau detection, after washing plates with the kit buffer, IBLpanTau(Fab’)HRP or IBLp181Tau(Fab’)HRP in ELISA kit was added into each well, then the plate was incubated at 4°C at 30 min.

After washing plates with PBS(−) for mAb005(Fab’)HRP or with the specified ice‐cold wash buffer for IBL detector antibodies (IBL kit component), fluorescent substrate of QuantaRed™ (Thermo Scientific, No. 15159) or colorimetric substrate TMB (1‐Step Ultra TMB‐ELISA) (Thermo Scientific, Cat. No. 34208) was added into each well. The plate was covered with aluminum foil to protect the reaction from light. Then, the enzymatically reacted substrate was read at *λ*ex/*λ*em of 570/585 nm with a band path of 5 nm for QuantaRed™ by microplate reader (Tecan, Safire II) or at OD450 nm for TMB by multimode plate reader (SpectraMax iD3, Molecular Device) after stopping the reaction with 1N phosphoric acid (Sigma Aldrich, Cat. No. 345245‐100 mL).

### 2.10 One‐site direct ELISA

The followings are conducted except described in two‐site sandwich ELISA. In brief, for HMWoTau specificity assay, various concentrations of each Aβ oligomer (Cat. No. SPR‐488), Aβ fibrils (Cat. No. SPR‐487), α‐synuclein oligomer (Cat. No. 484), or α‐synuclein fibrils (Cat. No. SPR‐322) (StressMarq) were added into each well (Fluotrac 96‐well black plate, high‐binding, Greiner, Cat. No. 655077) at protein contents from 1 to 1000 pg/well/100 μL in coating buffer A (100 mM Carbonate buffer, pH 9.6). For other studies of rhTau species or human brain tau species, samples were diluted by 400 times and above with coating buffer A. In particular, SDS/DTT treated samples were coated onto an ELISA plate at a final content of 30 pg. rhHMWoTau/well or 4 ng AD brain lysate protein/well after dilutions. After coating samples overnight at 4°C, plates were washed with PBS(−) and blocked with 1% (w/v) BlockAce in PBS(−). The plates were stored overnight at 4°C.

After washing plates with PBS(−), for comparison studies of recombinant protein, each well was treated at room temperature for 4 h with 100 μL of mAb005(Fab’)HRP (5000× dilution), mAb004(IgG)HRP (5000× dilution), BAN50‐biotin (100× dilution) in Aβ oligomer ELISA kit component (Fujifilm Wako, Cat No. 290‐82001) or D‐10 (IgG) (α‐synuclein monoclonal antibody, 0.5 μg/mL) (Santa Cruz, Cat No. sc‐515 879) in Intercept (PBS) Blocking Buffer. For human brain lysate studies, each well was treated with 100 μL of mAb005(Fab’)HRP (APRINOIA, 5000× dilution) overnight or IBLpanTau(Fab’)HRP (300× dilution) in PBS(−)/0.1%BSA/HAMA Blocking reagent for 30 min at 4°C.

For Aβ and α‐synuclein, after washing plates with PBS(−), each well was further treated with 100 μL of peroxidase‐conjugated streptavidin solution (100× dilution) (Aβ oligomer ELISA kit component) for Aβ or anti‐mouse IgG‐HRP (20 000× dilution) (Jackson ImmunoResearch, Cat. No. 115‐035‐146) for α‐synuclein in Intercept (PBS) Blocking Buffer. Then, the plates were incubated at room temperature for 1 h.

After washing all plates with PBS(−), except IBLpanTau(Fab’)HRP‐treated plate with IBL washing buffer, QuantaRed™ fluorescent substrate was added into each well. The enzymatically reacted substrate was read by a plate reader as described in two‐site‐sandwich ELISA.

### 2.11 Thioflavin S (Thio‐S) assay

The reacted sample or fractionated samples including HMWoTau were mixed with Thio‐S (Sigma, Cat. No. T1892‐25G) at a final concentration of 20 μM in PBS(−) in microplate (Fluotrac 96 well black plate, high‐binding, Greiner, Cat. No. 655077). The fluorescence intensity was read at *λ*ex/*λ*em of 440/485 nm with a bandwidth of 5 nm by microplate reader (Tecan, Sapphire II).

### 2.12 Immunological absorption using covalently crosslinked‐resin with normal mouse IgG, mAb005, mAb004, and HT7

Each normal mouse IgG (Fujifilm Wako, Cat. No. 140‐09511), mAb005(IgG), mAb004(IgG), and HT7(IgG) was covalently cross‐linked to the resin at a content of 10 μg using Pierce™ Crosslink IP Kit (Thermo Scientific, Cat. No. 26147), and immunological absorption of the sample was conducted according to the manufacture's instruction. The flow‐through fraction was used for ELISA to see the absorption effects. The immunologically absorbed mAb005(IgG)‐resin and normal mouse IgG‐resin labeled with Thio‐S were used for dSTORM Nanoimager analysis.

### 2.13 Atomic force micrography (AFM)

AFM data of HMWoTau were obtained using a Nano Wizard 4XP (Burker, Berine, Germany) combined with inverted microscope IX71 (Olympus, Tokyo, Japan) under Burker's QI™ (Quantitative Image) mode (about 10 ms/pixel) in PBS(−) buffer, using the cantilever “Peakforce‐HIRS‐F‐B” (Bruker, Camarillo, CA, USA) with typical spring constants of ~0.105 N/m. Purified HMWoTau solution (100 μL, 20 μg/mL) was allowed to adsorb for 10 min onto freshly cleaved mica pre‐coated with 0.01% poly‐l ornithine solution (Sigma, Cat. No. P4957). Then, the loaded mica was washed with PBS(−) buffer prior to AFM image acquisition. We processed the images of structures using JPK NanoWizard SPM and DP software (Burker, Camarillo, CA, USA). The diameter of HMWoTau was analyzed by NIH Image J (Version 1.54i) (https://imagej.net/ij/).

### 2.14 Super‐resolution microscopy using Nanoimager

Purified HMWoTau by SEC‐fractionation and the HMWoTau captured on normal mouse IgG‐ or mAb005(IgG)‐antibody‐resins were mixed with PBS(−) containing 20 μM of Thioflavin S. Each incubated sample was spotted on to slide glass and covered with a cover glass (Matsunami Glass Ind., Ltd., Japan) using VECTASHIELD® Antifade Mounting Medium (Vector Laboratories, H‐1000‐10).

Acquisition of super‐resolution image of HMWoTau was performed using dSTORM (direct Stochastic Optical Reconstruction Microscopy) of Nanoimager (Oxford Nanoimaging Limited (ONI), UK), equipped with an oil immersion objective (100×, 1.5 NA, UPLXAPO100XO, Evident, Japan) with 488 nm laser under the illumination angle set at 54°, detected by ORCA‐Flash 4.0 V3 digital CMOS camera (C12330‐20CU, Hamamatsu Photonics, Japan). This device generates structure image resolved to under 20 nm scale.

To obtain a final image of super‐resolved localizations of HMWoTau, 3000 images were obtained, under an exposure time of 30 ms and then subjected to NimOS software (Version:1.19.7, ONI, UK). The diameter of HMWoTau was analyzed by NIH Image J (Version 1.54i) (https://imagej.net/ij/).

### 2.15 Postmortem of human subjects

Human brain tissues of non‐Alzheimer's disease (NAD) and AD subjects were obtained from Banner Sun Health Research Institute (Sun City, AZ, USA),^47^ where brain pathological analysis of Aβ and tau had been completed, in advance of this study. All donor tissues were obtained with informed consent and used for studies under strict compliance with local and national IRB regulations as well as the Declaration of Helsinki. The demographic data are shown for individual subjects on Braak stage I (*N* = 12), II (*N* = 7), III (*N* = 5), IV (*N* = 5), V (*N* = 5) and VI (*N* = 6). For NAD and AD brain mixture studies, the selected age‐matched representatives were used as listed in Table 1 and in Table S1. For individual sample analysis, all subjects of S1 fractions were subjected to respective ELISAs (see all demographic data in Table 2). Data on ApoE genotype were not available for some subjects in Braak stage II and III. ApopE4 ratio was 17% for Braak stage I (*N* = 12), 60% for Braak stage IV (*N* = 5), 60% for Braak stage V (*N* = 5), and 50% for Braak stage VI (*N* = 6). Because of the small numbers of samples, the effects of APOE were not examined.

**TABLE 1:** Demographic data of representatives on AD and NAD subjects (*N* = 10).

No.	Braak stage	Age	Gender	PMI (h)	Apo E genotype
2	I	75	M	3.33	3/3
3	I	61	M	2.33	3/3
4	I	77	F	3.25	2/3
5	I	78	F	1.25	3/3
7	I	77	F	2.50	2/3
8	I	63	M	4.16	3/3
10	I	81	F	2.75	2/3
11	I	63	M	2.00	3/4
15	II	102	F	1.67	2/2
23	III	87	F	4.00	3/3
31	V	87	F	3.00	3/3
32	V	75	M	3.00	3/4
33	V	67	F	4.00	4/4
34	V	74	F	2.00	3/4
35	VI	81	F	2.25	4/4
36	VI	64	F	3.16	3/4
37	VI	75	M	2.25	3/3
38	VI	78	M	1.83	3/4
39	VI	76	F	2.00	3/3
40	VI	89	F	4.00	3/3

Abbreviations: F, female; M, male; PMI, postmortem interval.

**TABLE 2:** Demographic data of all subjects.

No.	Braak stage	Age	Gender	PMI (h)	Apo E genotype
1	I	53	M	3.63	3/3
2	I	75	M	3.33	3/3
3	I	61	M	2.33	3/3
4	I	77	F	3.25	2/3
5	I	78	F	1.25	3/3
6	I	70	M	3.00	2/4
7	I	77	F	2.50	2/3
8	I	63	M	4.16	3/3
9	I	82	M	1.66	3/3
10	I	81	F	2.75	2/3
11	I	63	M	2.00	3/4
12	I	81	M	2.75	3/3
13	II	80	M	3.25	3/3
14	II	85	M	2.50	2/3
15	II	102	F	1.67	2/2
16	II	82	M	2.83	3/3
17	II	83	M	4.50	N.A.
18	II	73	M	4.12	N.A.
19	II	79	M	1.66	3/3
20	III	74	M	4.58	N.A.
21	III	81	M	2.00	N.A.
22	III	75	M	2.00	3/3
23	III	87	F	4.00	3/3
24	III	68	M	2.00	3/3
25	IV	78	F	4.00	3/3
26	IV	78	M	3.33	3/4
27	IV	82	M	3.00	3/4
28	IV	77	M	3.25	2/3
29	IV	82	M	2.67	3/4
30	V	76	M	3.33	3/3
31	V	87	F	3.00	3/3
32	V	75	M	3.00	3/4
33	V	67	F	4.00	4/4
34	V	74	F	2.00	3/4
35	VI	81	F	2.25	4/4
36	VI	64	F	3.16	3/4
37	VI	75	M	2.25	3/3
38	VI	78	M	1.83	3/4
39	VI	76	F	2.00	3/3
40	VI	89	F	4.00	3/3

Abbreviations: F, female; M, male; PMI, postmortem interval.

### 2.16 Brain total lysate

Each human brain tissue was homogenized in a 5‐volume ice‐cold Tris‐based buffer (50 mM Tris, consisting of 2 mM EGTA and EDTA, 274 mM NaCl, 5 mM KCl) or PBS(−), containing protease inhibitors (cOmplete EDTA free, Roche, Cat. No. 1187358000120) and 1% phosphatase inhibitor cocktail II and III (Sigma, Cat. No. P‐5726 and P‐0044). The homogenate was centrifuged at 10 000 *g* for 15 min at 4°C to remove membranes and debris by Optima MAX‐XP Ultracentrifuge (Beckman Coulter), and the supernatant (S1) was collected for analysis in principle. To see the effects of gravity‐force and running time in centrifugation, the supernatant was further centrifuged at 100 000 g for 90 min, except as specified in the running‐time dependency study. The protein concentration was determined by Bradford protein assay (Quick Start Bradford 1× dye reagent, Cat. No. 5000205) using BSA as a standard (Takara, Japan, Cat. No. T9300A‐3) in principle except SEC study, where OD280 was determined using NanoDrop Lite (Thermo Scientific) to estimate protein levels after SEC. The protein concentration was expressed as mg/mL using BSA as a standard.

### 2.17 Statistical analysis

Two‐tailed student's *t* test was conducted for the comparison of two groups. Tukey's test or Dunnett's test was conducted for multiple comparisons as specified in the figures after ANOVA analysis using GraphPad Prism 8 software (San Diego, CA, USA). The data below background values of the standard were treated as “0”. The level of significance was defined as *p* < .05. For Tukey's test, the statistical difference was shown for the comparison between the control and other groups in each specified figure. All data are expressed as mean values ± SD in the text as well as figure legends.

## 3 RESULTS

### 3.1 Generation of rhHMWoTau detected by mAb005‐ELISA

It is essential to obtain calibrated standard proteins for quantitation using ELISAs in a reproducible and quantitative manner. We made efforts to generate recombinant human Tau (rhTau) (2N4R, full length)‐derived oligomer tau species because mAb005 was obtained using rhTau (2N4R) derived‐oligomers as immunogen components (submitted). We initially conducted a pilot study to find the best conditions to obtain the highest signal detected by mAb005‐mAb005(IgG)HRP ELISA among four groups of “rhTau alone,” “rhTau+heparin,” “rhTau+DTT,” and “rhTau+heparin+DTT” under the specified concentrations (Figure S1). The sandwich ELISA, consisting of the same capture and detector monoclonal antibody mAb005, should preclude the detection of monomer Tau by recognizing the same epitope on its molecular surface, and detecting tau oligomers with dimer and above, as done for other oligomer studies.^19^, ^46^ As a control study, Thioflavin S (Thio‐S) assay was conducted in parallel to monitor the formation of aggregated tau. The most efficiently formed tau‐aggregate was found in “rhTau+heparin.” The signals dramatically increased in both Thio‐S assay and ELISA in a time‐dependent manner. By contrast, both groups containing 2 mM DTT resulted in delay and/or inhibition of tau aggregate formation. It should be noted that the sustained increase was observed at 120 h for “rhTau+heparin” on the Thio‐S assay. By contrast, mAb005‐mAb005(IgG)HRP ELISA signal in “rhTau+heparin” showed a bell‐shaped change with peak levels at 24–48 h (Figure S1).

Tau aggregates/oligomers were reproducibly generated in PBS(−) at 37°C under the conditions of “rhTau + heparin” (Figure S2). Thio‐S assay showed the time‐dependent formation of tau aggregates with a peak at 48 h and leveled off thereafter (Figure S2A). The signal detected by mAb005‐mAb005(IgG)HRP ELISA reached a peak at an earlier time‐point of 24 h with a statistical significance and maintained the plateau level until 72 h. However, in agreement with a pilot study, the signal intensity tended to drop at 144 h with large variabilities (Figure S2B). In sum, the optimized incubation time for generation of target oligomer tau was concluded as 24–72 h.

Next, to examine heparin‐concentration dependency, we mixed rhTau (4 mg/mL diluted with PBS (−)) with various concentrations of heparin (10–640 μg/mL PBS(−)) and incubated the mixture at 37°C for 24 h. The results of Thio‐S assay (Figure S2C) and mAb005‐mAb005(IgG)HRP ELISA (Figure S2D) showed the same heparin concentration dependency with a statistical significance from 2X heparin (80 μg/mL), reaching a plateau at 8X heparin (320 μg/mL), being 7.4% of total protein concentration and above.

Based on the above optimization studies, we mixed rhTau (5.6 mg/mL) and a plateau level of heparin (650 μg/mL) (about 10% of total protein concentration) and incubated the mixture at 37°C for 3 days. The reaction mixture and non‐reaction mixture were separated by Size‐Exclusion‐Chromatography (SEC) to find the molecular size of the target protein. We employed “Superose 6 Increase 10/300 GL column” that has the fractionation range from 5 kD up to 5000 kD for globular proteins.

**FIGURE 1:**
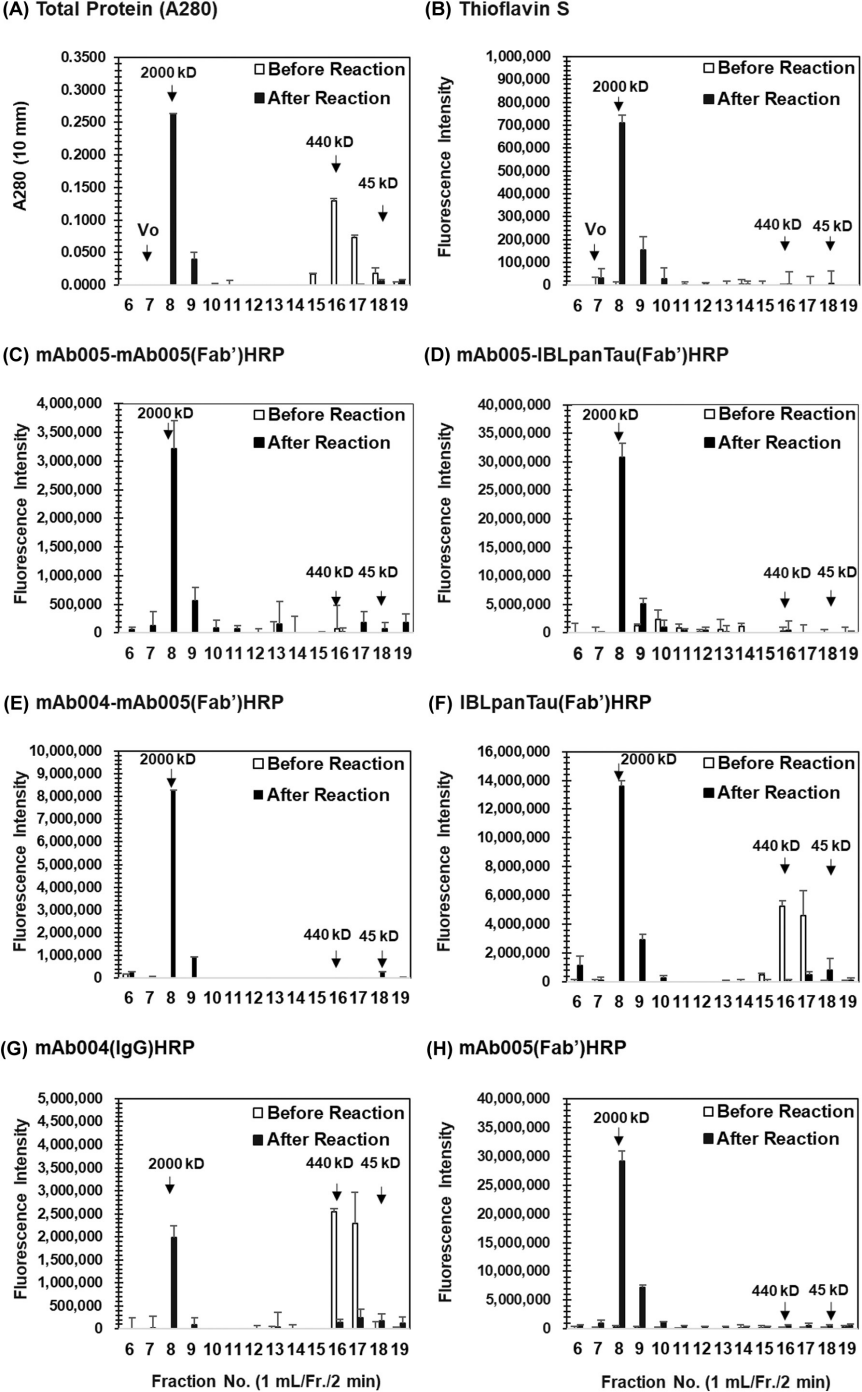
Selective detection of HMWoTau, but not MMWoTau/LMWoTau/mTau by Thioflavin‐S assay and mAb005‐ELISA. The reaction mixtures of rhTau (2N4R) and heparin or non‐reaction mixture were separated by Size‐Exclusion‐Chromatography (SEC) with Superose 6 Increase 10/300 GL column. Each fraction was subjected to (A) NanoDrop Light assay to determine total protein levels, (B) Thioflavin‐S assay to evaluate tau aggregates, a two‐sandwich ELISA of (C) mAb005‐mAb005(Fab’)HRP, (D) mAb005‐IBLpanTau(Fab’)HRP, and (E) mAb004‐mAb005(Fab’)HRP, one‐site direct ELISA of (F) IBLpanTau(Fab’)HRP, (G) mAb004(IgG)HRP (panTau in‐house antibody), and (H) mAb005(Fab’)HRP. Values are expressed as means ± SD (*N* = 3 determinations) after adjusted with background subtraction. Vo, void volume.

Probably due to too much high concentration of rhTau in stock solution (>10 mg/mL), total protein monitored by UV280 nm identified pre‐formed tau oligomers together with monomer Tau (mTau) at Fr. 15, 16, 17 and Fr. 18 (Figure 1A, open bar), named as middle‐molecular‐weight oligomer tau (MMWoTau) for Fr. 15 and 16 (between 670 and 158 kD), and as low‐molecular‐weight oligomer tau (LMWoTau) and mTau for Fr. 17 (about 158 kD and below), respectively. By contrast, almost all those peaks of pre‐formed oligomers/mTau disappeared after reaction and one‐single peak of high‐molecular‐weight oligomer Tau (HMWoTau) showed up at Fr. 8 (approximately 2000 kD tau species) next to void volume fraction 7 (Figure 1A, closed bar), being identified as a candidate of target molecule for mAb005‐ELISA. The absence of no protein in void volume fraction 7 indicates no further aggregated tau species are generated or the species might be trapped by column resins even if they were generated.

Then, each eluted sample was subjected to Thio‐S assay and mAb005‐mAb005(Fab’)HRP ELISA. Because we found a similar pattern of signal changes between Thio‐S assay (*λ*ex/*λ*em of 440/485) and mAb005‐mAb005(IgG)HRP ELISA in the time‐course study on tau‐aggregate formation (Figures S1, and S2), we considered that the reaction mixtures contained Thio‐S positive HMWoTau and/or just further aggregated Thio‐S positive tau species. However, we could not detect any Thio‐S positive signal at void volume Fr. 7 in SEC (Figure 1B), in consistent with protein distribution (Figure 1A). Therefore, we might fail to detect Thio‐S signal for further aggregated tau species in SEC fractionated samples.

Surprisingly, Fr. 8 sample (bearing circa 2000 kD) reacted with Thio‐S very strongly with a single peak (Figure 1B, closed bar). More Surprisingly, mAb005‐mAb005(Fab’)HRP ELISA also selectively detected the same peak signal at Fr. 8 (Figure 1C, closed bar). Because it is not clear if a single peak of HMWoTau could be detected by mAb005 alone, two types of two‐site sandwich ELISAs were additionally conducted, consisting of capture‐detector of mAb005‐IBLpanTau(Fab’)HRP (Figure 1D) and mAb004 (APRINOIA, N‐terminal epitope, in‐house pan‐tau antibody)‐mAb005(Fab’)HRP (Figure 1E). Interestingly, both mAb005‐ELISAs detected the same single peak signal only at Fr. 8, indicating that mAb005 itself is the determinant on detection of HMWoTau with circa 2000 kD.

To ensure if pan‐tau antibody of IBLpanTau and mAb004 are able to detect all Tau species, one‐site direct ELISA was set up for both pan‐antibodies. Then, all fractions were tested (Figure 1F,G, respectively). Both pan‐tau antibodies successfully detected all tau species of HMWoTau (Fr. 8), MMWoTau (Fri. 16), and LMWoTau/mTau (Fr. 17), indicating that both pan‐tau antibodies work to detect all tau species. To reconfirm mAb005's selectivity, one‐site direct ELISA of mAb005(Fab’)HRP was also set up and fractionated samples were tested (Figure 1H). Expectedly, mAb005(Fab’)HRP alone was enough to detect the same single peak signal at Fr. 8. Similar result was also obtained when one‐site direct ELISA of mAb005(IgG)HRP was conducted (data not shown).

All taken together, we concluded that rhHMWoTau (circa 2000 kD) located at Fr. 8 is the target molecule of mAb005. It was essential to use the optimized condition to obtain rhHMWoTau. Under other conditions such as different incubation time, protein concentrations of rhTau or heparin, and/or different buffer compositions, we failed to obtain a single peak and/or a large amount of HMWoTau at Fr. 8 (data not shown).

### 3.2 Morphological analysis of purified rhHMWoTau by AFM and Nanoimager

**FIGURE 2:**
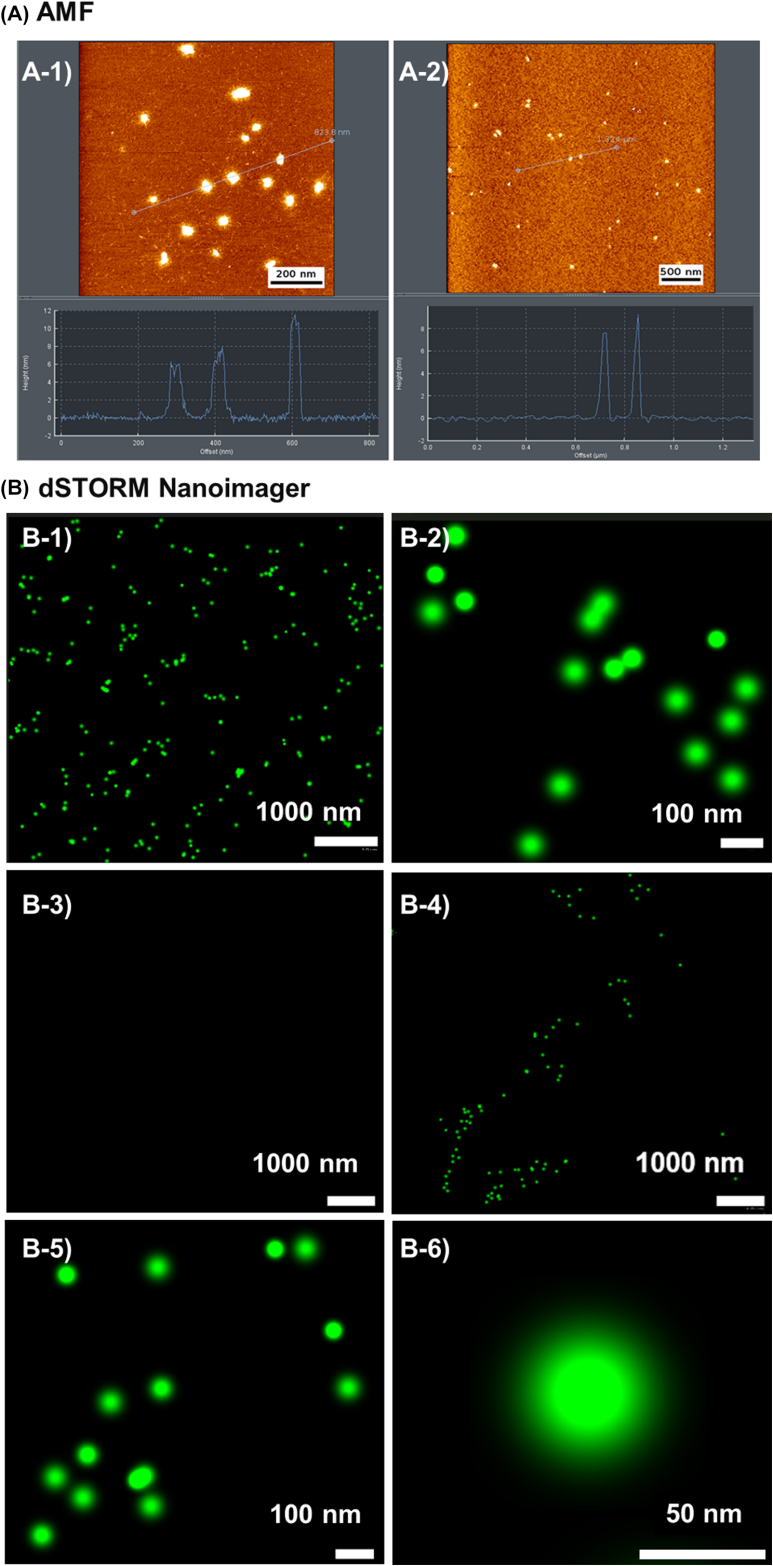
Visualization of globular oligomer particles in SEC‐purified sample of Fr. 8 by AFM and dSTORM‐Nanoimager. The molecules detected by mAb005‐ELISA at fraction 8 of SEC were subjected to (A) AFM analysis, showing globular oligomer form with an average diameter 31.8 ± 7.2 nm (*N* = 20) and about 10 nm (6–12 nm) height. The same fraction labeled with Thio‐S was further subjected to (B) dSTORM‐Nanoimager analysis, also revealing globular particles as shown in (B‐1) Scale bar; 1000 nm and in (B‐2) Scale bar; 100 nm. Estimated diameter was 31.9 ± 4.7 nm on average (*N* = 17). The same fraction 8 was immunologically captured by IgG resins. Then, the captured molecular species was labeled by Thio‐S and subjected to dSTORM‐Nanoimager analysis as shown in (B‐3) negative control normal mouse IgG‐resin, in (B‐4) mAb005(IgG)‐resin, Scale bar;1000 nm, in (B‐5) mAb005(IgG)‐resin, Scale = 100 nm. Estimated diameter was 34.8 ± 5.9 nm on average (*N* = 15) and in (B‐6) a representative image of the particle. Scale bar: 50 nm.

Because we found positive signals for HMWoTau (Fr. 8, circa 2000 kD) in both mAb005‐ELISA and Thio‐S assay, this fraction was further subjected to atomic force micrography (AFM) analysis to examine if Fr. 8 tau species have really homogeneous oligomeric particles or not (Figure 2A). Being similar to the molecular form in published articles,^42^, ^43^ all of our rhTau‐derived HMWoTau (Fr. 8 in SEC) showed only a globular particle with an average diameter of 31.8 ± 7.2 nm (*N* = 20) and an approximately 10 nm (6–12 nm) height. This molecular feature clearly indicates that Fr. 8's tau species does not contain any contaminated longer oligomers nor filamentous tau species.

To further confirm the above finding, we employed another technology of dSTORM (direct Stochastic Optical Reconstruction Microscopy). Nanoimager based on dSTORM can visualize molecules by scanning fluorescent‐labeled molecules also in physiological buffer conditions. Because we found rhHMWoTau in Fr. 8 was positive in Thio‐S assay (*λ*ex/*λ*em of 440/485), all tau species in Fr. 8 were incubated with Thio‐S (20 μM) and subjected to dSTORM analysis, resulted in the visualization of only homogeneous globular particles (Figure 2B‐1) with the same average diameter 31.9 ± 4.7 nm (*N* = 17) (Figure 2B‐2) as revealed by AFM study (Figure 2A). This result also ensured that Thio‐S positive molecules in Fr. 8 consist of globular oligomer particles without further aggregated tau species.

**FIGURE 3:**
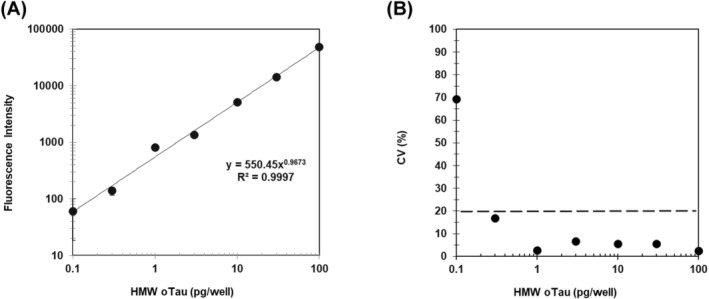
Standard curve for rhTau‐derived HMWoTau (rhHMWoTau) in a two‐site sandwich ELISA of mAb005‐mAb005(Fab’)HRP and CV plot. Various concentrations of HMWoTau were subjected to mAb005‐mAb005(Fab’)HRP ELISA. Capture antibody of mAb005 was coated into each well at 5 μg/mL (100 μL/well) and rhHMWoTau was detected by mAb005(Fab’)HRP. QuantaRed fluorescent substrate was used to detect ELISA signals by measurement at *λ*ex/*λ*em = 570/585 nm. (A) Standard Curve of mAb005‐mAb005(Fab’)HRP ELISA. Values are expressed as means ± SD (*N* = 3 determinations) after adjusted with background subtraction. SD error bars are within closed circles except 0.3 and 0.1 pg/well. (B) CV value for each concentration was plotted and LLOQ was determined.

**FIGURE 4:**
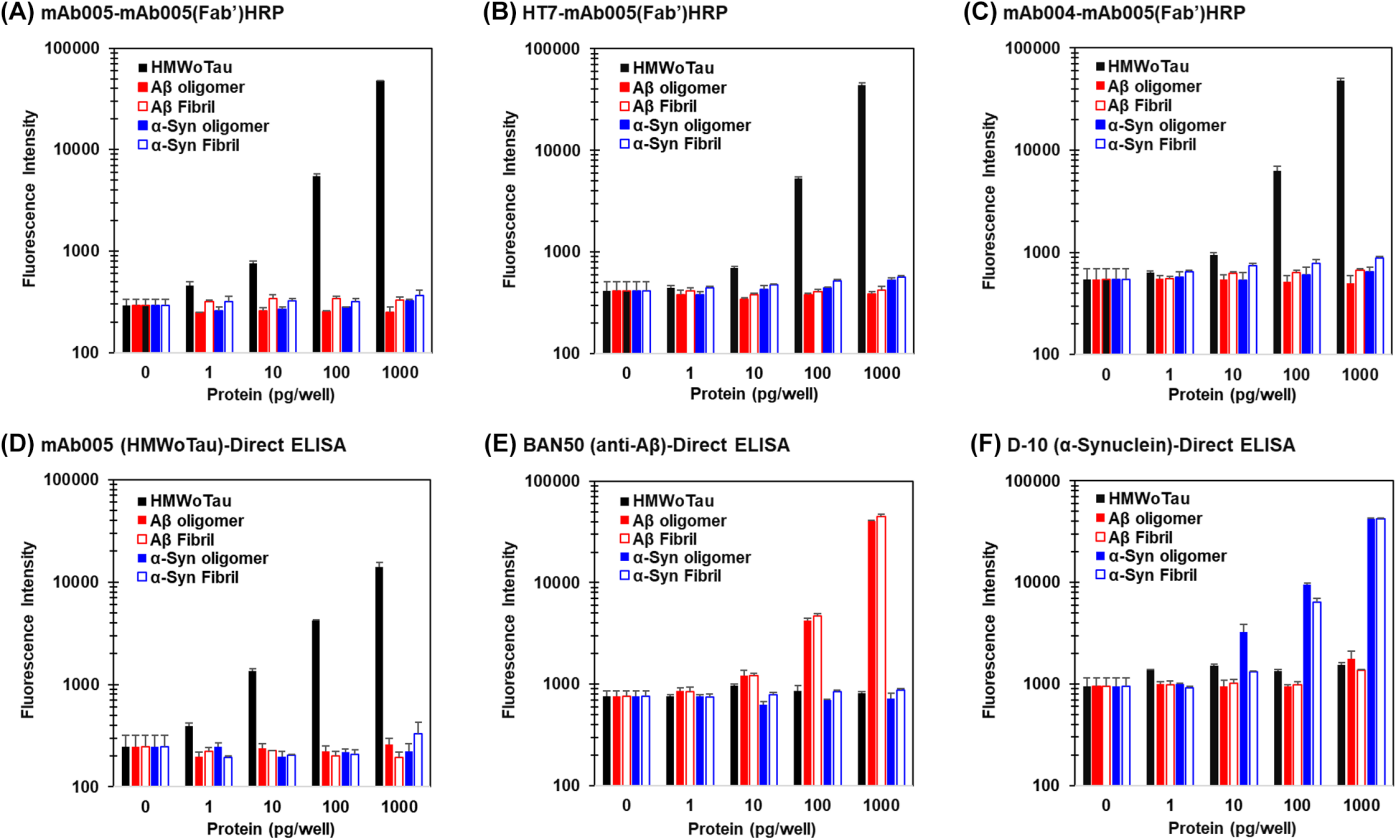
Selective recognition of rhHMWoTau by mAb005, without detecting other synthetic/recombinant‐derived human Aβ or α‐synuclein oligomer/fibrils in respective two‐site sandwich or one‐site direct ELISAs. Various concentrations of HMWoTau, Aβ oligomer/fibril, α‐synuclein oligomer/fibril were subjected to two‐site sandwich ELISAs consisting of three different capture antibodies (A) mAb005 (1 μg/mL), (B) pan‐Tau antibody HT‐7 (1 μg/mL), (C) another pan‐Tau antibody of mAb004 (1 μg/mL) in combination with a common detector antibody mAb005(Fab’)HRP. One‐site direct ELISA was conducted using the following antibody of mAb005, BAN50, and 10‐D monoclonal antibody for (D) HMWoTau, (E) Aβ oligomer/fibril, and (F) α‐synuclein oligomer/fibril, respectively. Values are expressed as means ± SD (*N* = 3 determinations) without adjustment by background subtraction.

**FIGURE 5:**
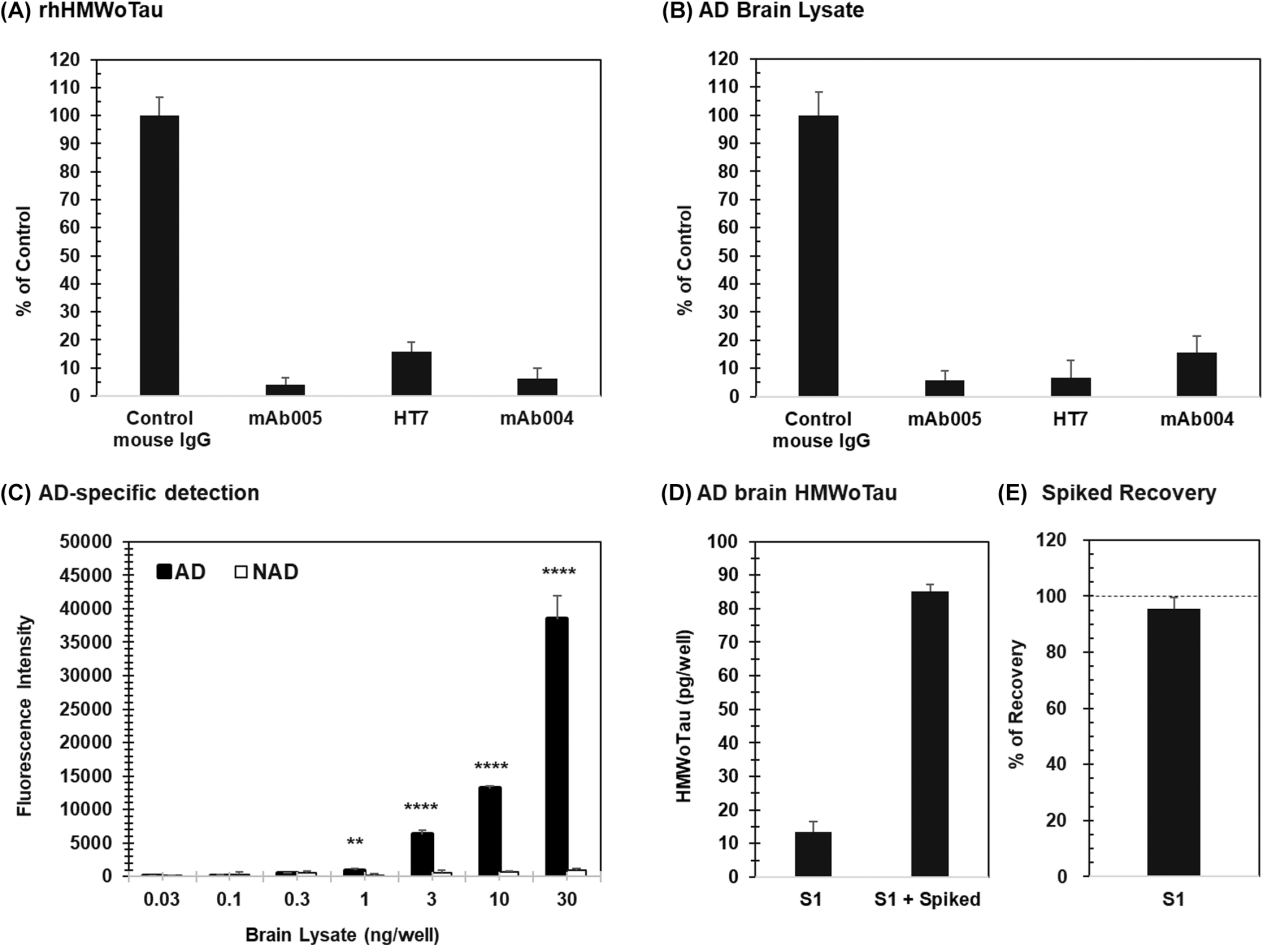
Specific quantitation of HMWoTau in human brain lysate (S1) by mAb005‐mAb005(Fab’)HRP ELISA was ensured by immunological absorption, concentration dependency on AD brain lysate specific detection and its spiked recovery. (A) Immunological absorption of rhHMWoTau (B) Immunological absorption of HMWoTau‐like molecules in AD brain lysate. (C) Total brain lysate protein‐dependent increase in HMWoTau‐like signal in AD. **p < .01, ***p < .001 versus NAD (Unpaired Student *t*‐test). (D) rhHMWoTau equivalent values in AD brain lysate with or without spiked rhHMWoTau. (E) Recovery of spiked rhHMWoTau in AD brain lysate. Values are expressed as means ± SD (*N* = 4 determinations).

**FIGURE 6:**
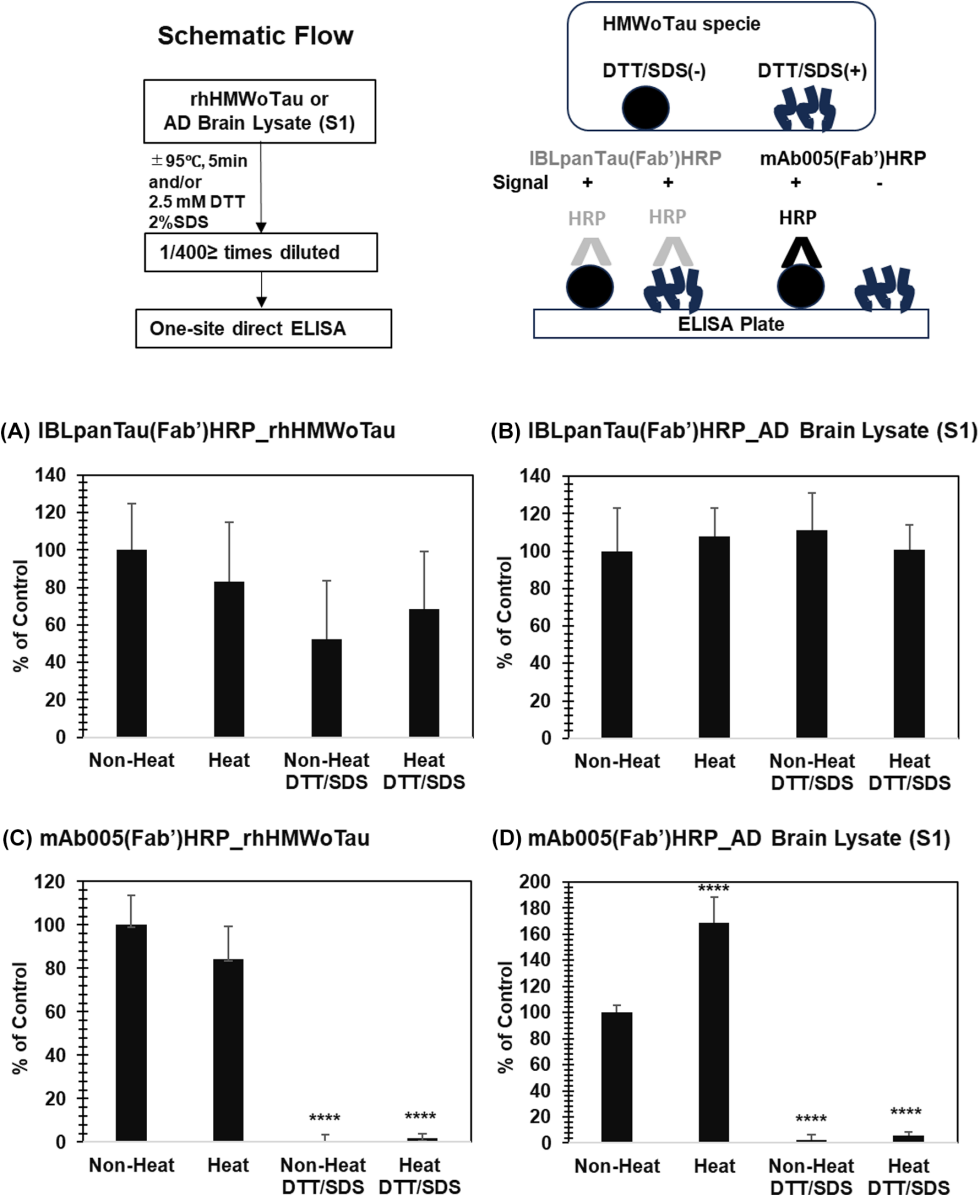
DTT/SDS treated HMWoTau was still recognized by panTau antibody, but not by mAb005. Recombinant human HMWoTau (rhHMWoTau) or AD brain lysate (S1) was pretreated with or without 2.5 mM DTT and 2% SDS under heat‐denature or non‐heat‐denature conditions as illustrated (Left). Then, the samples, diluted by 400 times and above, were subjected to one‐site direct ELISA of IBLpanTau(Fab’)HRP for (A) rhHMWoTau and (B) AD brain lysate, respectively, or one‐site direct ELISA of Ab005(Fab’)HRP for (C) rhHMWoTau and (D) AD brain lysate, respectively, as illustrated (Right). Values are means ± SD (*N* = 6 determinations). ****p < .0001 versus Non‐Heat (Tukey's test).

To get direct evidences that the target molecule of mAb005 is globular oligomer tau species, we did the immunological absorption study with mAb005(IgG)‐resin, using normal mouse IgG‐resin as a control. The absorbed resin materials were labeled with Thio‐S (20 μM) and subjected to Nanoimager, resulting in the detection of only globular particles in mAb005‐resin, being almost the same average diameter of 34.8 ± 5.9 nm (*N* = 15) (Figure 2B‐4, 5, 6). By contrast, negative control of normal mouse IgG‐resin did not show any of those globular particles (Figure 2B‐3), eliminating IgG‐resin's non‐specific absorption. Magnified images with totally spherical forms are shown in Figure 2B‐5 (sale bar = 100 nm) and Figure 2B‐6 (scale bar = 50 nm, a representative image).

In sum, by two independent morphological analysis methods, we confirmed that the target molecule of mAb005 in Fr. 8 of SEC is HMWoTau consisting of only homogenous globular particles with an average diameter of about 30 nm, without any contamination of further aggregated tau species.

### 3.3 High sensitivity to detect recombinant human (rh) Tau‐derived HMWoTau in mAb005‐mAb005(Fab’)HRP ELISA

After optimization of mAb005‐mAb005(Fab’)HRP ELISA, we could successfully obtain rhHMWoTau standard curve with quite a good linearity (*R* ^2^ = 0.9997) (Figure 3A). The LLOQ was estimated as 0.3 pg/well/100 μL (3 pg/mL). The minimum concentrations' coefficient of variation (CV) was <20% at 0.3 pg/well, being used as a LLOQ criterion (Figure 3B). In optimization study, the detection limit was dramatically improved by approximately 100 times, compared with that of colorimetric TMB substrate that is often used in traditional ELISA (see Figure S3).

### 3.4 mAb005 is specific to HMWoTau, but not to monomer tau nor to other protein aggregate species of Aβ/α‐synuclein

We observed no detection of monomer Tau (mTau) detected by mAb005‐ELISAs at Fr. 17 of SEC as demonstrated in Figure 1C–E,H. To confirm no detection of mTau by mAb005, we compared our standard rhHMWoTau and commercially available monomer tau (IBL, Japan, Cat. No. 50163) under clinically measurable range of concentrations (10, 100, and 1000 pg/mL) in highly sensitive sandwich ELISAs as follows: (1) total Tau ELISA of mAb004‐IBLpanTau(Fab’)HRP, (2) HMWoTau ELISA of mAb004‐mAb005(Fab’)HRP. We found the concentration‐dependent increase in both species of HMWoTau and mTau standard proteins in mAb004‐IBLpanTau(Fab’)HRP ELISA as expected (Figure S4A). By contrast, we could detect HMWoTau signals selectively in a concentration‐dependent manner in mAb004‐mAb005(Fab’)HRP ELISA (Figure S4B), ensuring the mAb005's specificity to HMWoTau, but not to mTau. No recognition of mTau was also confirmed using a combination study with mAb005‐ELISAs and Sucrose‐Density‐Gradient‐Centrifugation (SDGC) (see result section of Figure 9).

Next, under the same protein concentrations (1–1000 pg/well/100 μL = 10–10 000 pg/mL), we further compared rhHMWoTau and other protein species oligomers and fibrils of Aβ or α‐synuclein that are commercially available as validated proteins from StressMarq. We used ELISAs consisting of the capture of mAb005, pan‐tau antibody HT7 as well as mAb004 in combination with a common detector mAb005(Fab’)HRP (Figure 4). These all mAb005‐ELISA pairs showed a concentration‐dependent and selective increase in the signal of HMWoTau, but not of other Aβ/α‐synuclein oligomers/fibrils (Figure 4A–C). To make sure if each protein species really works in ELISA or not, respective oligomers and fibrils species of Aβ and α‐synuclein, together with HMWoTau, were also subjected to one‐site direct ELISA consisting of each specific detector antibody of clone mAb005 for HMWoTau, clone BAN50 for Aβ and clone 10‐D for α‐synuclein. We observed a concentration‐dependent and specific increase in respective signals as expected (Figure 4D–F). All taken together, these results confirmed the specificity of mAb005 antibody to HMWoTau.

### 3.5 mAb005 detected AD‐specific ELISA signals in human brain lysate mixtures

mAb005 clone has been discovered as AD‐specific tau antibody. To make sure of the mAb005‐ELISA's specificity, immune‐absorption study of AD brain lysate was conducted, using our purified rhHMWoTau as a positive control (Figure 5A). Pretreatment of rhHMWoTau with cross‐linked mAb005(IgG)‐, mAb004(IgG)‐, and HT7(IgG)‐resins successfully reduced those mAb005‐mAb005(Fab’)HRP ELISA signals, compared with that of control normal mouse IgG‐resin, indicating rhHMWoTau molecule bears respective tau epitopes on its molecular surface such as N‐terminal tau residues for mAb004 and 159–163 tau residues for HT7, besides the epitopes of mAb005.^40^

Under this head‐to‐head comparison study, the pretreatment of AD brain lysate with the above‐mentioned respective tau antibody (mAb005, HT7, and mAb004) IgG‐resins also decreased mAb005‐mAb005(Fab’)HRP ELISA signal selectively (Figure 5B), indicating AD brain‐derived HMWoTau species have the same epitopes on the target molecule in common. We have obtained further supporting results in studies in one‐site direct ELISA of mAb005(Fab’)HRP as well as a two‐site ELISA of mAb004‐mAb005(Fab’)HRP on these immune‐absorption effects (data not shown).

Next, we examined the dilution dependency of mAb005's signal using AD and NAD brain lysate mixtures in mAb005‐mAb005(Fab’)HRP ELISA. AD‐specific signal was observed in a statistically significant manner from 1 ng total brain lysate protein/well (**p < .01 vs. NAD, unpaired student *t*‐test) and above in a dilution‐dependent manner, without any signal detection in NAD (Figure 5C).

We also conducted a spiked recovery study using rhHMWoTau as a standard in mAb005‐ELISA, which resulted in a full recovery of more than 95% (Figure 5D,E).

All taken together, we confirmed the specific and quantitative measurement of HMWoTau species in AD brain lysate in a very highly sensitive manner from 1 ng total brain lysate protein/well in mAb005‐ELISA.

### 3.6 Conformation‐specific recognition of HMWoTau species by mAb005

DTT and SDS have been used in WB analysis, as known to break S–S bond and to disrupt 3D‐structure/complex form of proteins to be changed into liner and disassemble proteins. If mAb005 is a conformation‐specific antibody, the mAb005‐ELISA signal should disappear by treatment of samples with DTT/SDS. Here, rhHMWoTau and AD brain lysate (S1) were treated with or without 2.5 mM DTT and 2% SDS under non‐heat (on‐ice) or heat‐denature conditions. Then, the diluted samples (by 400 times and above) were subjected to one‐site direct ELISAs consisting of mAb005(Fab’)HRP or IBLpanTau(Fab’)HRP (Figure 6).

We found that “DTT/SDS” treatment did not statistically change the signals detected by one‐site direct ELISA of panTau(Fab’)HRP, irrespective of heat‐denature, not only on rhTau‐derived HMWoTau (Figure 6A), but also on HMWoTau species in AD brain lysate (Figure 6B). These results indicate that pan‐tau antibody (IBLpanTau(Fab’)) is not a conformation‐specific antibody for both rhHMWoTau and AD‐derived HMWoTau species.

By contrast, “DTT/SDS” treatment reduced the signals detected by one‐site direct ELISA of mAb005(Fab’)HRP in a statistically significant manner, irrespective of heat‐denature, not only on rhTau‐derived HMWoTau (Figure 6C) but also on HMWoTau species in AD brain lysate, in common (Figure 6D) (****p < .0001 vs. Non‐Heat, Tukey's test). Similar results were also observed in a two‐site sandwich ELISA of mAb005‐mAb005(Fab’)HRP and mAb004‐mAb005(Fab’)HRP for both rhHMWoTau and AD‐derived HMWoTau species (data now shown). There was no influence of DTT/SDS under 400 times diluted conditions on samples as confirmed by a standard curve of HMWoTau and monomer Tau in mAb005‐ and total Tau ELISA (data not shown).

In sum, mAb005 antibody is concluded as a conformation‐specific antibody to both rhHMWoTau and AD brain lysate‐derived HMWoTau species, in common.

### 3.7 Characterization of HMWoTau species in AD brain lysate by centrifugation‐based fractionation

**FIGURE 7:**
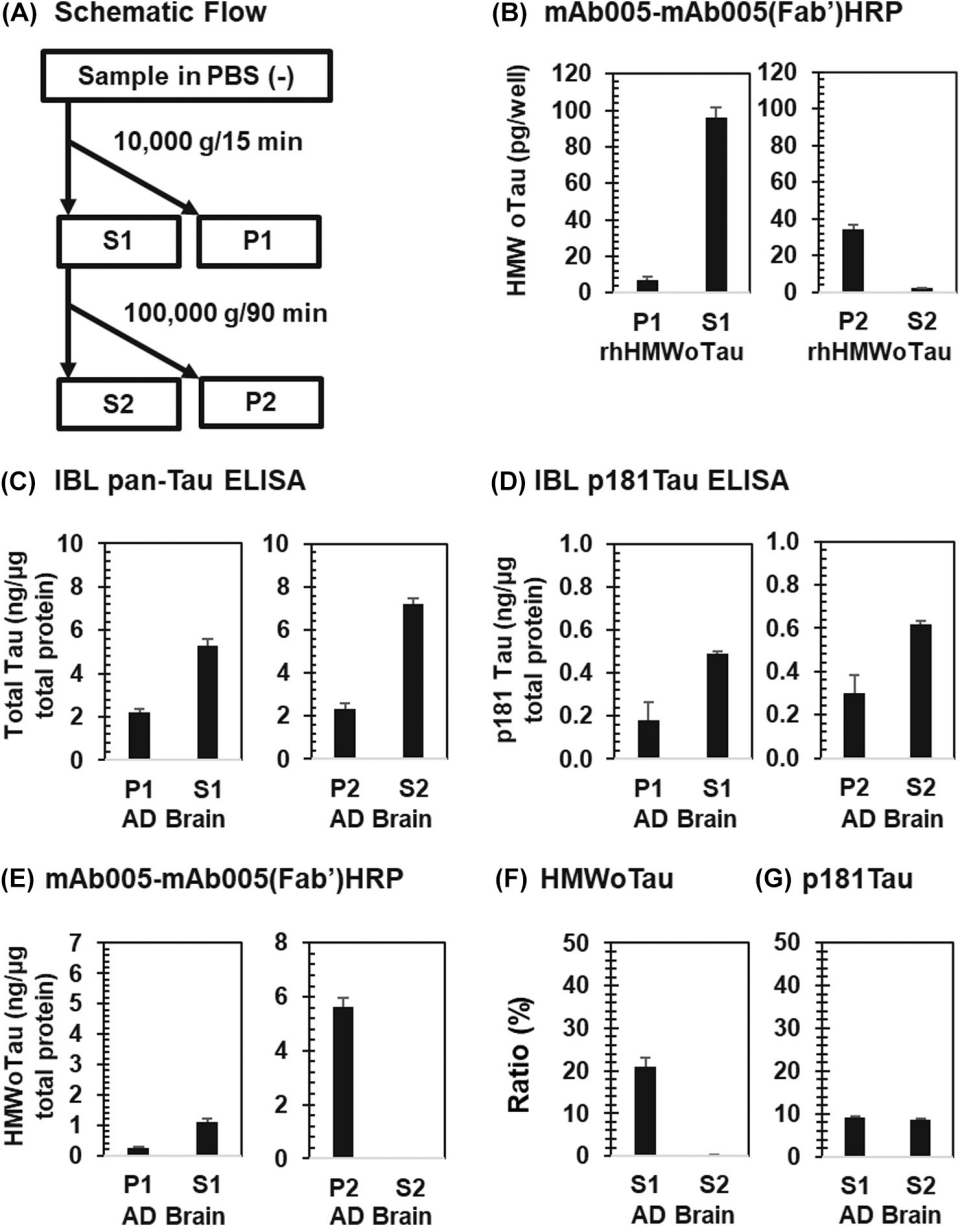
Ultracentrifugation with 100 000 *g* (90 min) precipitated HMWoTau species for both rhTau‐derived and AD brain lysate‐derived HMWoTau. (A) Schematic flow of centrifugation‐based fractionation of recombinant human HMWoTau (rhHMWoTau) and AD brain lysate protein mixtures. The fractionated samples of rhHMWoTau were subjected to a two‐site sandwich ELISA of (B) mAb005‐mAb005(Fab’)HRP ELISA. The fractionated samples of AD brain lysate were subjected to two‐site sandwich ELISA of (C) Total Tau ELISA, (D) p181Tau ELISA, and (E) mAb005‐mAb005(Fab’)HRP ELISA. Ratio (%) was shown on (F) HMWoTau to total Tau and (G) p181Tau to total Tau for AD brain lysate samples. Values are means ± SD (*N* = 3 determinations).

It is essential to identify in which fraction HMWoTau species will be collected by centrifugation for the quantitation of HMWoTau by ELISA. Thus, the step‐wise fractionation was conducted as illustrated (Figure 7A). We focused on S1 and S2, because the pellet was sonicated with PBS(−) containing 0.01% Tween 20, maybe leading to (i) artificial changes such as disruption of native form of structure and/or (ii) further extraction of HMWoTau/relevant tau species from membrane/lipid particles on human samples.

Purified rhTau‐derived HMWoTau (rhHMWoTau) was used as a positive control. The sample is centrifuged at 10 000 *g* for 15 min in PBS(−), resulting in the localization of rhHMWoTau into S1 with more than 90% (Figure 7B, Left). The further ultracentrifugation of S1 at 100 000 *g* for 90 min eliminated the HMWoTau signal in S2 and only pellet 2 (P2) showed the positive signal, while the recovery seems to be low (Figure 7B, Right).

In head‐to‐head comparison, human AD brain lysates were also evaluated. In the beginning, the levels of total Tau and p181Tau were measured as reference tau biomarkers using commercially available IBL total Tau and p181Tau kits (Figure 7C,D). Both total Tau and p181Tau were dominantly collected into S1 and S2 after centrifugation at 10 000 *g* (15 min) and at 100 000 *g* (90 min), respectively. By contrast, being similar to rhHMWoTau (Figure 7B), human brain‐derived HMWoTau species was found mainly in S1 after 10 000 *g* (15 min) (Figure 7E, Left). Then, ultracentrifugation at 100 000 *g* (90 min) completely eliminated the signal from S2 and positive signals were only found in P2 (Figure 7E, Right). As expected and described above, P2 fraction of AD showed a higher amount of HMWoTau species by about 6 times of S1 source after sonication of the pellet using PBS(−) containing 0.01% Tween 20. It should be noted that almost no signal is present in S2. However, in order to get this clear result, it was very critical and essential to run ultracentrifugation for 90 min and above, this is because the shorter running time of ultracentrifugation at 100 000 *g* insufficiently precipitate HMWoTau species into P2 (Figure S5).

**FIGURE 8:**
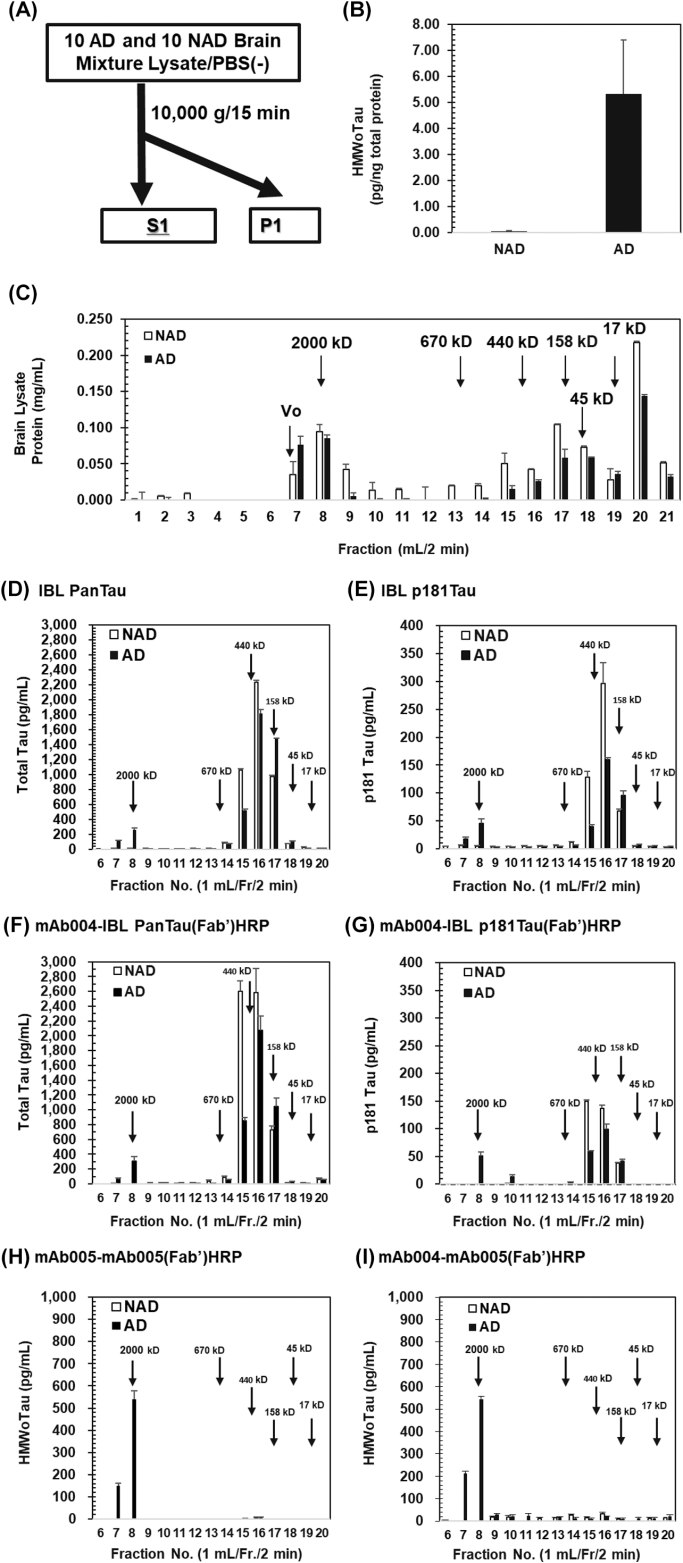
Selective detection of HMWoTau, but not MMWoTau/LMWoTau/mTau in Size‐Exclusion‐Chromatography (SEC) fractionated samples by mAb005‐ELISAs. (A) Schematic procedure of sample preparation for SEC fractionation, (B) HMWoTau levels before fractionation of respective AD and NAD brain lysate (S1). Values are expressed as means ± SD (*N* = 10 subjects). (C) Fractionated AD and NAD brain mixtures by SEC with Superose 6 Increase 10/300 GL column. Fractionated protein concentration was expressed as mg/mL using BSA as a standard after quantitation by NanoDrop Lite. Values are means ± SD (*N* = 3 determinations). Each 100 times diluted fraction was subjected to the following ELISA of (D) IBL total Tau kit, (E) IBL p181 Tau kit, (F) mAb004‐IBLpanTau(Fab’)HRP ELISA, (G) mAb004‐IBLp181Tau(Fab’)HRP ELISA, (H) mAb005‐mAb005(Fab’)HRP ELISA, and (I) mAb004‐mAb005(Fab’)HRP ELISA. Values are expressed as means ± SD (*N* = 3 determinations). Vo, void volume.

When the ratio of HMWoTau molecules to total Tau was calculated on each S1 and S2 for AD brain lysate mixtures, HMWoTau occupied approximately 20% and 0% in S1 and S2 fraction, respectively (Figure 7F). By contrast, p181Tau occupied approximately 10% in both S1 and S2 fractions (Figure 8G).

In sum, the target molecule of mAb005‐ELISA in PBS(−)‐extractable AD brain lysate was dominantly fractionated into S1 after low‐speed centrifugation (10 000 *g*, 15 min), but not into S2 after ultracentrifugation (100 000 *g*, 90 min), as commonly observed also in rhHMWoTau. This profile is different from those of total Tau and p181Tau species. Thus, a common source of PBS(−)‐extractable S1 fraction was used for further analysis of HMWoTau, total Tau, and p181Tau throughout this report, except specified elsewhere.

### 3.8 mAb005 recognized AD brain lysate‐derived HMWoTau species at the same fraction 8 in SEC as observed in rhHMWoTau

Because mAb005‐ELISA's target molecule was identified as HMWoTau in the rhTau‐derived oligomer mixtures by combination analysis with SEC and mAb005‐ELISA (Figure 1), the target molecule in AD brain lysates could be also HMWoTau. Thus, we evaluated the AD brain lysate mixture (S1) in a similar manner to that analysis of rhHMWoTau, in comparison with NAD brain lysate.

In the beginning, each S1 sample from age‐matched subjects was obtained from AD (*N* = 10, Age; 76.6 ± 7.4 y) and NAD (*N* = 10, Age; 76.4 ± 11.8 y) as illustrated in Figure 8A (demographic data are shown in Table 1). We confirmed AD‐specific detection of HMWoTau‐like signal by mAb005‐mAb005(Fab’)HRP ELISA. The average value was 5.32 ± 2.09 ng/μg total brain lysate protein for AD, under detection limit levels of 0.04 ± 0.03 ng/μg for NAD (Figure 8B). Equal protein concentration of S1 lysate was mixed to prepare the respective NAD and AD brain lysate mixture.

The equal amount of NAD and AD brain mixtures was then subjected to SEC equipped with Superose 6 Increase 10/300 GL column (fractionation range of globular proteins; 5–5000 kD). Distribution analysis of protein concentration for fractionated samples revealed that higher molecular weight (HMW) proteins than 2000 kD were observed at Fr. 7 (void volume), particularly slightly higher in AD, implying AD has more aggregated proteins than that of NAD (Figure 8C). AD brain lysate showed equivalent protein levels between Fr. 7 and Fr. 8 as well as between Fr. 17 and Fr. 18 (Figure 8C, closed bar). By contrast, NAD brain lysate showed high peaks of protein level at Fr. 8 and Fr. 17 (Figure 8C, open bar). These fractionated samples were subjected to respective two site‐sandwich ELISAs of total Tau (IBL total Tau ELISA, mAb004‐IBLpanTau(Fab’)HRP ELISA), p181Tau (IBL p181 ELISA, mAb004‐IBLp181Tau(Fab’)HRP ELISA), and HMWoTau (mAb005‐mAb005(Fab’)HRP ELISA, mAb004‐mAb005(Fab’)HRP ELISA) using each of recombinant human tau‐derived standards. All ELISAs showed quite a good linearity with *R* ^2^ values > 0.99 for each standard curve (see each standard curve in Figure S6). Each spiked standard's recovery rate was >90% (data not shown).

Firstly, the levels of total Tau and p181Tau were analyzed by IBL ELISA kits, respectively (Figure 8D,E). Being different from the total brain lysate protein's separation pattern, the ELISA signals of Fr. 8 were observed only in AD (closed bar) for both total Tau and p181Tau, with values of about 264 ± 18 and 45 ± 8 pg/mL (*N* = 3 determinations), respectively. It should be noted that the levels of total brain lysate protein were almost the same between NAD and AD at Fr. 8, indicating AD selective increase in HMWoTau species of total Tau and p181Tau at Fr. 8.

NAD showed higher levels of total Tau than those of AD at both Fr. 15 and Fr. 16 corresponding to MMWoTau (located between 670 and 150 kD) (AD; 519 ± 16 vs. NAD; 1058 ± 24 pg/mL, ****p < .0001 at Fr. 15, AD; 1821 ± 49 vs. NAD; 2234 ± 29 pg/mL, **p < .01 at Fr. 16, unpaired Student's *t*‐test) (Figure 8D). NAD also showed higher levels of p181 Tau than those of AD at both Fr. 15 and Fr. 16 (AD; 40 ± 4 vs. NAD; 129 ± 10 pg/mL, ****p < .0001 at Fr. 15, AD; 159 ± 5 vs. NAD; 296 ± 37 pg/mL, ***p < .01 at Fr. 16) (Figure 8E). However, both Fr. 15 and Fr. 16 had also statistically higher protein content in NAD than in AD (*p < .05, ***p < .01 at Fr. 15 and Fr. 16) (Figure 8C).

By contrast, AD showed higher levels of LMWoTau (dimer)/mTau located at Fr. 17 detected by IBL total Tau ELISA (AD; 1476 ± 10 vs. NAD; 972 ± 17 pg/mL, ****p < .0001) (Figure 8D) and by IBL p181Tau ELISA (AD; 96 ± 8 vs. NAD; 67 ± 4 pg/mL, **p < .01, unpaired Student's *t*‐test) (Figure 8E), in spite of statistically lower levels of total brain lysate protein in AD than NAD (***p < .001) (Figure 8C, closed bar), indicating the levels of total Tau and p181Tau detected by IBL kits substantially increased in AD at Fr. 17.

To confirm the above‐mentioned results, we conducted another set of a two‐site sandwich ELISA of total Tau and p181Tau by replacing the IBL capture antibody (anti hTau441‐E22A3 rat IgG monoclonal antibody) with mAb004 (Figure 8F,G).

Consistently, we found the similar pattern of tau species to those observed in Figure 8D,E. The levels of total Tau and p181Tau at Fr. 8 were similar levels of 350 ± 2 and 51 ± 7 pg/mL, respectively, to those obtained by commercially available IBL kits (Figure 8D,E). NAD brain lysate of Fr. 15 (MMWoTau species) also showed higher levels of total Tau (AD; 852 ± 45 vs. NAD; 2602 ± 137 pg/mL, ****p < .0001) and of p181Tau (AD; 59 ± 1 vs. NAD; 150 ± 2 pg/mL, ****p < .0001). p181Tau also showed statistically higher values at Fr. 16 in NAD than in AD (AD; 99 ± 9 vs. NAD; 137 ± 6 pg/mL, **p < .01).

By contrast, the tau species of LMWoTau (dimer) or mTau located at Fr. 17 also showed slightly but statistically higher levels of total Tau in AD than in NAD (AD; 1050 ± 114 vs. NAD; 730 ± 51 pg/mL, *p < .05) (Figure 8F), as observed in IBL total Tau ELISA. However, there was no difference in p181Tau level (Figure 8G).

There were some differences in absolute values between IBL's commercial kits and its modified ELISA with mAb004 as a capture antibody. Total Tau of NAD in modified ELISA (2602 ± 137 pg/mL) was higher than that in IBL kits (1058 ± 24 pg/mL) at Fr. 15. p181Tau of NAD in modified ELISA (137 ± 6 pg/mL) was lower than that in IBL kits (296 ± 37 pg/mL), suggesting some capture differences between two antibodies of IBL's E22A3 clone and mAb004 clone.

Next, we examined HMWoTau species level using two different mAb005‐ELISA; one is mAb005‐mAb005(Fab’)HRP ELISA and another is mAb004‐mAb005(Fab’)HRP ELISA. As expected, we found HMWoTau signal only in AD for both ELISAs with a peak at Fr. 8 (MW of circa 2000 kD) of a similar level of 540 ± 37 pg/mL (Figure 8H) and 545 ± 13 pg/mL (Figure 8I), respectively. It should be noted that NAD lysates did not show any signal in all fractions in both mAb005‐ELISAs, in spite of the presence of abundant levels of MMWoTau/LMWoTau as well as mTau, as revealed by total Tau and p181Tau ELISAs (Figure 8D–G).

We found mAb005 is the determinant to detect HMWoTau species in AD by comparison of Figure 8F and Figure 8I, where the difference is the detector antibody of either IBLpanTau (Fab’)HRP or mAb005(Fab’)HRP under the same pan‐tau capture antibody of mAb004. Together with our finding to detect rhHMWoTau by mAb005‐ELISA in rhTau‐derived tau species (Figure 1), these human brain lysates data indicate mAb005 is a bona fide specific antibody to HMWoTau (circa 2000 kD) species located in Fr. 8 for both rhHMWoTau and AD brain lysate‐derived HMWoTau species, in common. We also should pay attention to the fact that the levels of MMWoTau species between 670 and 158 kD (Fr. 15 and Fr. 16) are not higher in AD than in NAD as demonstrated by two different sets of total Tau ELISAs and p181Tau ELISAs.

In order to learn (1) the difference in oligomer tau antibodies between mAb005 and commercially available and well‐known T22 and (2) the contribution of more advanced Braak stage to detect HMWoTau species, we prepared another set of human brain lysate mixtures, consisting of age‐matched NAD subjects only from Braak stage I (*N* = 6, Age; 76.0 ± 6.9) and AD subjects with more ratio of Braak stage VI (*N* = 6, Age; 77.5 ± 9.2, Braak stage V = 2 subjects, Braak stage VI = 4 subjects) (see Table S1).

Each of the NAD and AD brain mixtures showed similar levels of total lysate protein at Fr. 13–19 between AD and NAD, except Fr. 7 and Fr. 8 in which AD only showed higher amount of total lysate protein (Figure S7A). The levels of Fr. 7 and Fr. 8 in AD were almost the same protein concentration (about 0.1 mg/mL). This indicates that proteins with high molecular weight appear later than Braak stage I, in comparison with the data of NAD consisting of Braak stage I, II, and III (Figure 8C).

Under these total lysate protein distributions, we examined the levels of total Tau and p181Tau and found robustly increased levels of HMWoTau species detected by both total Tau ELISA and p181Tau ELISA at Fr. 8 (Figure S7B,C, respectively), being above 30–50 times more than those in 10 AD and 10 NAD subjects (Figure 8F,G).

By contrast, the levels of MMWoTau and LMWoTau/mTau (Fr. 15–17) detected by total Tau ELISA and p181Tau ELISA (Figure S7B,C) were about two times higher than those observed in the cohort of 10 AD and 10 NAD subjects (Figure 8D–G). In this new cohort, NAD reproducibly showed statistically increased levels of MMWoTau detected by total Tau ELISA than those of AD at Fr. 16 only (AD; 2428 ± 223 vs. NAD; 5163 ± 482 pg/mL, **p < .001), while total brain lysate protein was also higher in NAD than AD. By contrast, there were no statistical differences between NAD and AD on p181Tau level at Fr. 16 and Fr. 17.

HMWoTau species detected by mAb005‐ELISAs also increased robustly with similar range levels (about 1500–2600 pg/mL) in three different mAb005‐ELISAs; (1) mAb005‐mAb005(Fab’)HRP, (2) mAb004‐mAb005(Fab’)HRP, and (3) Ab005‐IBLpanTau(Fab’)HRP ELISA (Figure S7D–F). The levels were 3–5 times higher than those in 10 AD and 10 NAD subjects (about 500 pg/mL, Figure 8H,I).

All taken together, these results indicate more advanced AD generated more HMWoTau species detected at Fr. 8, not only by mAb005‐ELISAs but also more robustly by total Tau ELISA as well as p181Tau ELISA.

T22, a polyclonal oligomer tau antibody, is commercially available and has been traditionally used in many articles to detect oligomer Tau species.^15^, ^28^, ^48^ Here, the difference between mAb005 and T22 to capture tau oligomer species was compared using a two‐site sandwich ELISA under the same detector of IBLpanTau(Fab’)HRP. In consistent with the previous reports, T22 showed multiple oligomers' peaks. However, we have shown more clearly that not only Fr. 14–17 but also Fr. 8 had substantial oligomer signals. T22 could also recognize rhTau‐derived HMWoTau with a similar standard curve to that of mAb005‐ELISA (data not shown). The HMWoTau level detected by T22 at Fr. 8 in AD showed a similar level (about 2000 pg/mL) to that of mAb005‐ELISAs (Figure S7G). In sum, T22 antibody is concluded as an antibody for multiple oligomers, being different from the profile of mAb005.

### 3.9 mAb005‐ELISA recognized not only HMWoTau as a minimum size of oligomer but also a longer form of tau oligomer species, as demonstrated by Sucrose‐Density‐Gradient‐Centrifugation (SDGC) and ELISAs for both rhTau‐ and AD brain lysate‐derived tau species

Because Superose 6 Increase 10/300 GL column used in SEC has a limitation of fractionation range (5–5000 kD of globular protein), we are unable to evaluate if mAb005 can recognize such higher MW size of tau aggregates species beyond 5000 kD, or not. Thus, we adopted Sucrose‐Density‐Gradient Centrifugation (SDGC) that has been utilized to separate monomer tau and various oligomer tau species including HMWoTau (circa 2000 kD) as well as filamentous tau aggregates in earlier reports.^42^, ^43^

**FIGURE 9:**
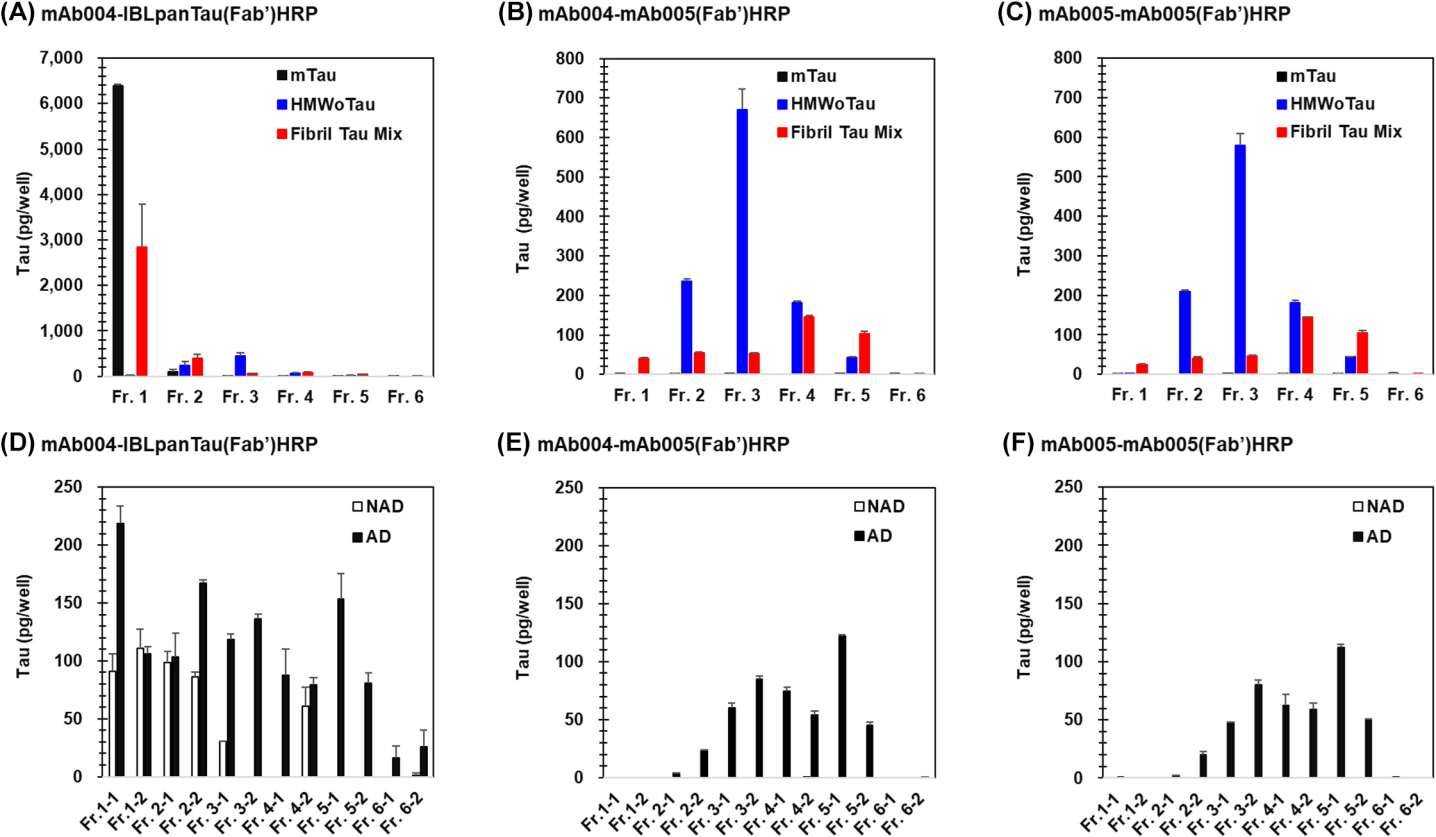
Specific mAb005's detection of HMWoTau and more oligomerized forms by ELISA analysis in combination with Sucrose‐Density‐Gradient‐Centrifugation (SDGC) of both rhTau‐derived and human brain lysate derived‐tau species. For recombinant tau proteins, an equal amount (20 μg/200 μL) of tau protein species of monomer tau, HMWoTau, or tau fibril mixture was fractionated by SDGC. Each fraction of 1000 μL from top to bottom was collected. For human brain lysate (S1), an equal amount (400 μg/200 μL) of AD or NAD brain lysate mixture was fractionated by SDGC. Each fraction of 500 μL from top to bottom was collected. Diluted fraction samples were subjected to (A, D) mAb004‐IBLpanTau(Fab’)HRP ELISA, (B, E) mAb004‐mAb005(Fab’)HRP ELISA, or (C, F) mAb005‐mAb005(Fab’)HRP ELISA. Values are expressed as tau protein (pg/well) with means ± SD of 3 determinations.

In the beginning, we examined if three different recombinant tau‐derived samples, consisting of (1) monomer Tau, (2) HMWoTau, and (3) aggregated Tau mixtures, could be separated successfully or not, using various tau ELIASs as follows: total Tau ELISA consisting of mAb004‐IBLpanTau(Fab’)HRP ELISA to identify all tau protein species (Figure 9A). Then, two different mAb005‐ELISAs: mAb004‐mAb005(Fab’)HRP (Figure 9B) and mAb005‐mAb005(Fab’)HRP (Figure 9C) to detect HMWoTau signals.

Total Tau ELISA showed the highest signal for monomer tau at Fr. 1 (Sucrose 10% w/v) with about 6400 pg/well (Figure 9A, black closed bar). By contrast, purified rhHMWoTau from Fr. 8 in SEC was selectively collected into Fr. 3 (Sucrose 30% w/v) with a single peak detected by total Tau ELISA (455 ± 68 pg/well) (Figure 9A, blue closed bar). There were no other peak fractions such as Fr. 1 (monomer Tau), Fr. 2 (LMWoTau, MMWoTau), and Fr. 4, 5, and 6 (longer oligomerized tau and filamentous tau aggregates),^43^ indicating that Fr. 8 in SEC (circa 2000 kD) did not contain higher oligomers/fibrils beyond 2000 kD. Our finding was in line with the reported localization at Fr. 3 for both HMWoTau species derived from AD brain and rhTau.^42^, ^43^ By contrast, Tau‐fibril mixtures were localized from Fr. 1 to Fr. 6 (pellet) (Figure 9A, red closed bar), with still high content of monomer tau (about 2842 ± 945 pg/well) at Fr. 1, then at Fr. 2 (about 392 ± 91 pg/well), and followed by a smaller but substantial amount of more oligomerized tau species at Fr. 4 (about 87 ± 19 pg/well). Under these total tau species' distributions, we examined how mAb005‐ELISAs could detect each of those tau species.

Both mAb005‐mAb005(Fab’)HRP and mAb004‐mAb005(Fab’)HRP commonly detected purified rhHMWoTau at fraction 3 (30%) with a single peak (670 ± 53 pg/well and 580 ± 30 pg/well, respectively) (Figure 9B,C, blue closed bar), being consistent with the similar level detected by total Tau ELISA (Figure 9A, blue closed bar), ensuring again that rhTau‐derived HMWoTau is a single molecular species of HMWoTau bearing circa 2000 kD.

By contrast, tau aggregate mixtures showed peaks at Fr. 4 with about 150 pg/well, and then at Fr. 5 with about 100 pg/well in both mAb005‐ELISAs (Figure 9B,C, red closed bar). These results indicate that mAb005 is able to bind the artificially generated tau aggregates located in Fr. 4 and Fr. 5. And mAb005 itself is the determinant to detect not only HMWoTau but also more oligomerized forms. Both mAb005‐ELISAs did not detect any signals of mTau fractionated into Fr. 1 (Figure 9B,C, black closed bar), in agreement with SEC result, where there was no signal at Fr. 17 (corresponding to dimer/mTau) (Figure 1C–E,H).

Next, we evaluated PBS(−)‐extractable S1 fractions of NAD and AD brain lysate mixtures in a similar manner. Total Tau ELISA signals were observed in both AD and NAD brain lysates in common except no signal of NAD at Fr. 3‐2, Fr. 4‐1, Fr. 5‐1, Fr. 5‐2, Fr. 6‐1, and Fr. 6‐2 in these cohorts (Figure 9D). The tau species at Fr. 1‐2, 2‐1, and 4‐2 were almost equally distributed between AD and NAD, and the tau species were higher in AD at Fr. 1‐1, 2‐2, 3‐1, 3‐2, 4‐1, 5‐1, 5‐2, 6‐1, and 6‐2.

Under these total tau species distributions, both mAb005‐ELISAs of mAb004‐mAb005Fab'HRP (Figure 9E) and mAb005‐mAb005Fab'HRP (Figure 9F), showed almost the same detection pattern with two main peaks at Fr. 3‐2 (about 80 pg/well) and at Fr. 5‐1 (about 120 pg/well). No signals were detected in both mAb005‐ELISAs for all fractions of NAD samples (Figure 9E,F) in spite of the substantial presence of those total Tau species as revealed by total Tau ELISA (Figure 9D). It should be noted that Fr. 4‐2 in NAD, containing more oligomerized tau species than HMWoTau (circa 2000 kD), was strongly detected by total Tau ELISA, but not by mAb005‐ELISAs at all. This is in line with no detectable levels of mAb005‐ELISAs for NAD brain mixtures in total brain lysate (Figures 5C and 8B).

In sum, mAb005 is concluded as a specific antibody against HMWoTau (circa 2000 kD) as a minimum size of oligomer tau species, together with its more oligomerized tau species (Fr. 5‐1) in AD, but not the more oligomerized tau species (Fr. 4‐2) in NAD brain lysate (S1). Hereafter, the term “HMWoTau species” was used for both a minimum size of tau oligomer “HMWoTau” (circa 2000 kD) and its more oligomerized tau species with a peak at Fr. 5‐1.

### 3.10 Both total Tau and p181Tau statistically increased at Braak stage V and VI under their substantial amounts at Braak stage I–III, in PBS(−)‐extractable brain lysates (S1) from the frontal lobe

We further analyzed the levels of tau species in S1 lysate derived from pathologically confirmed brain of the frontal lobe, consisting of Braak stage I (*N* = 12), II (*N* = 7), III (*N* = 5), IV (*N* = 5), V (*N* = 5), and VI (*N* = 6) as shown in the demographic data (Table 2). The subjects of Braak stage V and VI are clinically and pathologically diagnosed with AD.

**FIGURE 10:**
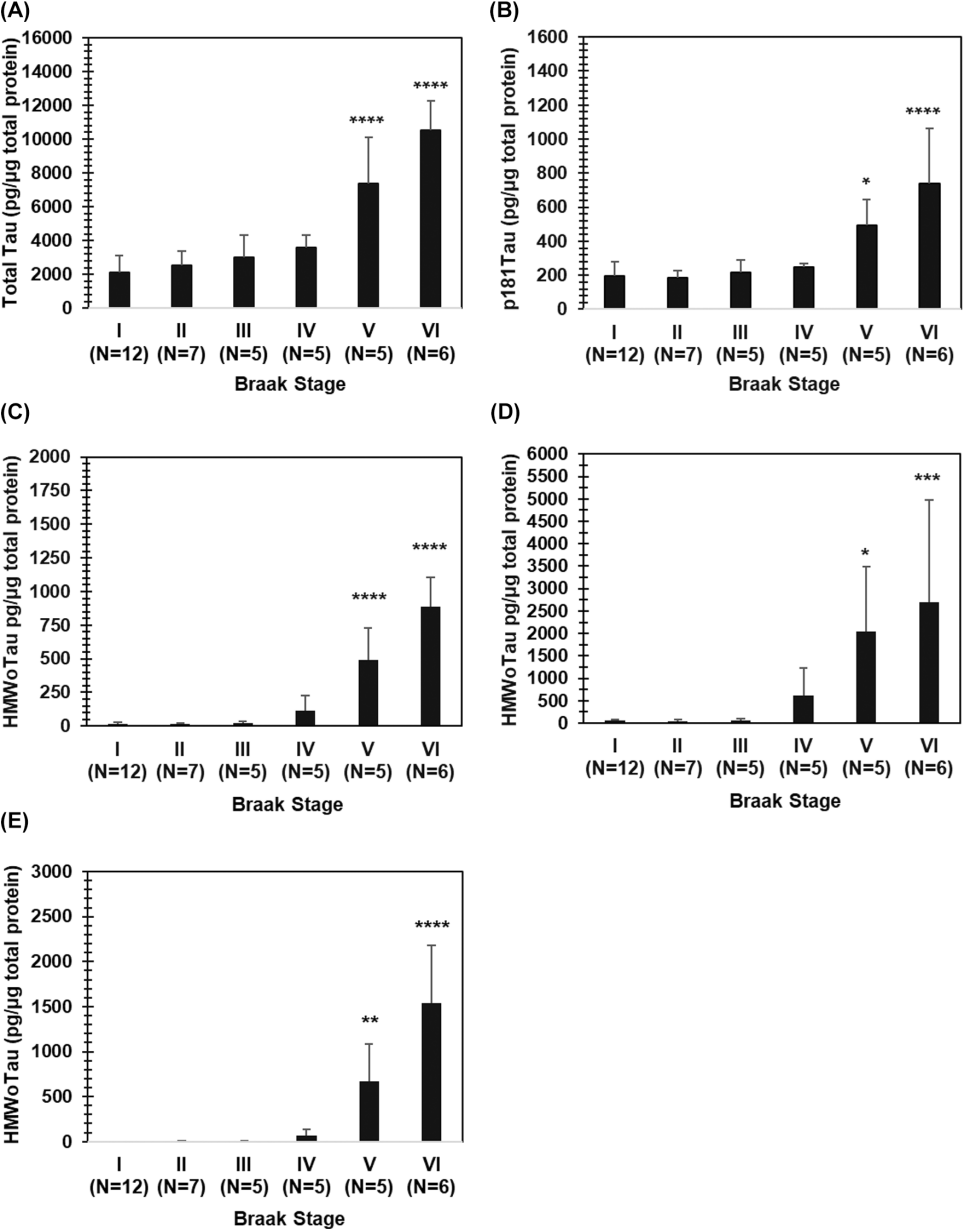
Statistically significant increase in total Tau, p181Tau, and HMWoTau species at Braak stages V and VI. Each human brain lysate (S1) sample was subjected to total Tau ELISA of (A) mAb004‐IBLpanTau(Fab’)HRP, p181Tau ELISA of (B) mAb004‐IBLp181Tau(Fab’)HRP, two different HMWoTau‐ELISAs of (C) mAb005‐mAb005(Fab’)HRP and (D) mAb004‐mAb005(Fab’)HRP or one‐site direct ELISA of (E) mAb005(Fab’)HRP. Values are expressed as means ± SD (*N* = 5–12 subjects, as specified). *p < 0.05, **p < .01, ***p < .001, ****p < .0001 versus Braak Stage I, Tukey's test.

To clarify the profile of all tau species' levels, we quantitated the levels of total Tau by mAb004‐IBLpanTau(Fab’)HRP ELISA and p181Tau by mAb004‐IBLp181Tau (Fab’)HRP ELISA, respectively. Interestingly, compared with the control level of total Tau at Braak stage I (2109 ± 1006 pg/μg total brain lysate protein), a statistically significant increase was observed at Braak stage V (7364 ± 2729 pg/μg, ****p < .0001) as well as at Braak stage VI (10 505 ± 1765 pg/μg total protein, ****p < .0001) (Tukey's test) (Figure 10A). Similarly, compared with the control level of p181Tau (195 ± 83 pg/μg), a statistically significant increase was observed at Braak stage V (493 ± 154 pg/μg, *p < .05) as well as at Braak stage VI (741 ± 321 pg/μg, ****p < .0001) (Figure 10B). Both tau species were present substantially at Braak stage I, being 1/4–1/5 fold of those of Braak stage VI.

### 3.11 HMWoTau species drastically increased at Braak stages V and VI, compared with the detection limit levels of Braak stage I–III, in PBS(−)‐extractable brain lysates (S1) from the frontal lobe

We quantitated the levels of HMWoTau species by a two‐site sandwich ELISA of mAb005‐mAb005(Fab’)HRP and mAb004‐mAb005(Fab’)HRP.

HMWoTau species detected by mAb005‐mAb005(Fab’)HRP ELISA showed a drastic increase in Braak‐stage dependent manner, substantially from Braak stage IV, and with a statistical significance at Braak stage V (492 ± 234 pg/μg total protein, ****p < .0001) and Braak stage VI (888 ± 219 pg/μg, ****p < .0001), compared with the detection limit levels of Braak stage I (18 ± 11 pg/μg). Average values for Braak stage I–III were almost the same values of 18–25 pg/μg (Figure 10C). HMWoTau species detected by mAb004‐mAb005(Fab’)HRP ELISA also showed a substantial increase from Braak stage IV, and statistically significant and drastic increases at Braak stage V (2040 ± 1441 pg/μg, *p < .05) and Braak stage VI (2703 ± 2271 pg/μg, ***p < .001), compared with Braak stage I (52 ± 36 pg/μg total protein). Average values for Braak stage I–III were also similarly low levels of 52–60 pg/μg total protein (Figure 10D). The average values were higher than those of mAb005‐mAb005(Fab’)HRP ELISA while SD was also larger, probably due to capture with pan‐tau antibody mAb004. Fold difference in HMWoTau species between Braak stage I and Braak stage VI was approximately 50 times for both mAb005‐ELISAs.

We further conducted one‐site direct ELISA using mAb005(Fab’)HRP alone. Interestingly, similar statistically significant levels of HMWoTau were observed at Braak stage V and VI, with values of 667 ± 419 and 1541 ± 640 pg/μg total protein, respectively (Figure 10E), ensuring the above‐mentioned Braak‐staged dependent increase detected by a two‐site sandwich ELISA of mAb005.

## 4 DISCUSSION

As far as we know, this is the first report of a novel ELISA to quantitate HMWoTau species with molecular weight (MW) of circa 2000 kD as a minimum size for both rhTau‐derived and human brain lysate‐ derived tau species.

In addition, we also demonstrated for the first time that HMWoTau species increased in Braak stage‐dependent manner on PBS(−)‐extractable fraction S1 (10 000 *g*, 15 min) in the frontal lobe, substantially at Braak stage IV, and in a statistically significant and robust manner at Braak stage V and VI of AD, compared with the detection limit levels of NAD, indicating their disease‐specific generation.

mAb005‐ELISA could quantitate HMWoTau levels at a clinically measurable range of concentrations from LLOQ of 3 pg/mL (0.3 pg/well) up to 1000 pg/mL (100 pg/well) and above, in a highly sensitive and specific manner (Figure 3A, Figure S3A). LLOQ of 3 pg/mL is comparable or superior to those of commercially available tau‐related ELISA kits based on the following kit information: (1) p181Tau ELISA kits such as Innotest®Phospho‐Tau(181P) (Fujirebio, Cat. No. 81581, detection limit = 13 pg/mL) and human p181Tau assay kit‐IBL (IBL, Cat. No. 50171, detection limit = 17.2 pg/mL). (2) Total Tau ELISA kits such as Innotest®hTau Ag (Fujirebio, Cat. No. 81579, detection limit = 34 pg/mL) and human total tau assay kit‐IBL (IBL, Cat. No. 50161, detection limit = 4.31 pg/mL). Thus, our highly sensitive ELISA will be useful not only as a research tool but also for potential diagnostics. In fact, mAb005‐ELISA showed the specific signal in AD at a total lysate protein level of 1 ng/well and above (Figure 5C). This ng level detection by mAb005‐ELISA is different by 1000 times and above, compared with the levels being used in western blot analysis at 1–20 μg/well of total lysate protein.

Total Tau and p181Tau levels also statistically increased at Braak stages V and VI, compared with those substantial levels at Braak stage I. It should be noted that the levels of NAD (Braak stages I–III) occupied 1/4–1/5 levels of Braak stage VI of AD, implying their physiological roles of total Tau and p181Tau in NAD. We should also underscore that these total Tau and p181Tau molecular species in brain lysate (S1) consist of not only monomer full‐length Tau/p181 Tau (Fr. 17 in SEC) but also multiple oligomer species as observed such as HMWoTau (Fr. 8), MMWoTau (Fr. 15, 16) in SEC (Figure 8, Figure S7) as well as various tau oligomer/aggregate species at Fr. 2, 3, 4, 5, and 6 in SDGC (Figure 9). The HMWoTau level (Fr. 8) detected by total Tau ELISA showed a similar level to that of mAb005‐ELISA (Figure 8). However, the amount exceeded the level detected by mAb005‐ELISA in the more advanced Braak stage of the cohort (Figure S7). Thus, the drastic increase in total Tau and p181Tau at Braak stage V and VI in brain lysate (S1) (Figure 10A,B) could be partly explained by the robustly increased HMWoTau species at Fr. 8 in SEC. We need to continue further studies to elucidate those disease‐associated tau oligomer species from etiological view points, including abnormally phosphorylated tau species besides p181Tau.

We successfully generated recombinant human Tau (rhTau)‐derived HMWoTau as a standard of mAb005‐ELISA. The optimized condition was crucial and essential to obtain a single peak with homogenous globular particles of HMWoTau at Fr. 8 (circa 2000 kD) in SEC (Figures 1 and 2). It was important to monitor HMWoTau levels with mAb005‐ELISAs (Figures S1 and S2), in combination with SEC. When incubation‐time, each concentration of rhTau and heparin and/or ratio as well as buffer compositions were changed, we failed to obtain a single peak of HMWoTau at Fr. 8. Instead, we observed multiple peaks of oligomer Tau species and/or very low amount of HMWoTau species in SEC study, or highly aggregated tau species were found in pellet even by low‐speed centrifugation (10 000 *g*, 15 min) (unpublished observations). Through these experiences, we realize many kinds of tau aggregates have been generated in past studies.

We could eliminate the possibility that Fr. 8 in SEC (Superose 6 Increase) might contain more aggregated tau species besides HMWoTau (circa 2000 kD) derived from rhTau (2N4R) because of the following three evidences, at least.

1. The rhHMWoTau species in Fr. 8 consisted of only globular oligomer particles, bearing an average diameter of about 30 nm (31.8 ± 7.2 nm), without detection of longer filamentous tau species (Figure 2A), as clearly demonstrated by AFM analysis that has been used to visualize various tau oligomer/aggregate forms in fractions of SDGC.^43^
2. Another technology of dSTORM (direct Stochastic Optical Reconstruction Microscopy)‐based Nanoimager elucidated that the same rhHMWoTau species of Fr. 8 in SEC consists of a homogeneous globular oligomer particle labeled with Thio‐S, in agreement with the same average diameter obtained by AFM; about 30 nm (31.9 ± 4.7 nm), without detection of any other longer forms of tau aggregate species (Figure 2B). The technology of dSTORM has been also used in the field of neurodegenerative disease study to visualize various forms of aggregated Aβ species.^49^
3. The same rhHMWoTau species of Fr. 8 in SEC was further separated by Sucrose‐Density‐Gradient‐Centrifugation (SDGC) to isolate various tau oligomer/aggregate species based on molecular size, mass, and buoyant density, used for separation of various tau species,^43^ resulted in a single peak at Fr. 3 in all tau‐ELISAs in common (Figure 9A–C, blue closed bar). This fraction of tau species corresponds to globular HMWoTau with a molecular weight of circa 2000 kD as reported. If our purified rhHMWoTau contains longer oligomers/filamentous tau species, we should also find another peak at a higher sucrose density fraction above Fr. 3. We actually detected rhTau‐derived longer oligomer than HMWoTau for the sample of tau aggregated mixtures at Fr. 4 and Fr. 5 by mAb005‐ELISA (Figure 9B,C, red closed bar). These aggregates did not show up as a peak in SDGC for our purified rhHMWoTau (Figure 9B,C, blue closed bar).

Regarding AD brain derived HMWoTau species, we found a single peak signal of mAb005‐ELISAs only at Fr. 8, in combination analysis with Tau ELISAs and SEC (Superose 6 Increase). We did not observe another equivalent or higher mAb005‐ELISA peak at Fr. 7 (void volume) to be associated with a similar level of the total lysate protein to that of Fr. 8 (Figure 8C) or with the higher level of total lysate protein at Fr. 7 (Figure S7A). These results suggest that AD oligomer Tau species fractionated at Fr. 8 in SEC mainly consists of HMWoTau bearing MW of circa 2000 kD.

However, the highest fractionation range of molecular weight on SEC (Superose 6 Increase column) is circa 5000 kD, being technically difficult to separate and analyze such higher molecular species beyond 5000 kD. Thus, we extended to examine human brain lysate mixtures, in combination analysis with Tau ELISAs and Sucrose‐Density‐Gradient‐Centrifugation (SDGC) that enables to separate highly aggregated tau species in an elegant manner.^43^

As expected, we found more oligomerized Tau species are abundantly present in AD brain lysate (S1) above Fr. 3 (corresponding to circa 2000 kD) as well as in NAD brain lysate (S1) at Fr. 4–2 (>2000 kD), as revealed by total Tau ELISA (Figure 9D). Two different mAb005‐ELISAs revealed the target tau species not only in the peak at Fr. 3 but also another peak at Fr. 5‐1 only in AD (Figure 9E,F, black closed bar). The presence of HMWoTau (circa 2000 kD) at Fr. 3 in SDGC was in line with HMWoTau as reported.^43^ Another more oligomerized Tau species of Fr. 5‐1 was considered to have about 7‐fold longer length (circa 200 nm on average; 198 ± 174 nm) than that of HMWoTau (c.a. 30 nm), by estimation with the picture in the report^43^ using NIH Image J. These results indicate that mAb005 selectively binds oligomerized species located in Fr. 3 and Fr. 5‐1 in SDGC fractionation, but does not bind NAD‐derived oligomer species located at Fr. 4‐2, bearing longer length than HMWoTau (Fr. 3).^43^

We could not obtain the evidence that SEC (Superose 6 increase column) was able to successfully separate the more oligomerized Tau species than HMWoTau (circa 2000 kD), corresponding to Fr. 4‐2 in NAD and Fr. 5‐1 in AD after SDGC fractionation (Figure 9D). We see Fr. 5‐1's slightly higher level of tau signals than that of Fr. 3's HMWoTau in AD lysate after SDGC fractionation. If the exact amount of more oligomerized Tau species corresponding to AD's Fr. 5‐1 after SDGC could be successfully fractionated by SEC, the potent Tau ELISA signals should be also detected at void volume fraction 7, as actually an equivalent or higher total lysate protein was evidently detected in AD at Fr. 7 in SEC (Figure 8C, Figure S7A). In addition, if the higher molecular weight of oligomer species corresponding to NAD's Fr. 4‐2 after SDGC could be successfully fractionated by SEC, the potent total Tau ELISA signal should be also detected at Fr. 7 of void volume fraction or Fr. 8. However, we could not observe any such signals for NAD brain lysate, clearly indicating that the highly oligomerized tau species not only corresponding to Fr. 4‐2 (NAD) but also to Fr. 5‐1 (AD) failed to be fractionated in SEC. The reason could be due to the trap of those highly oligomerized tau species by column resins (Superose 6 Increase) or pretreatment of the sample with re‐centrifugation before SEC for column protection.

It should be also noted that there was no mAb005‐ELISA signal at pellet fractions (Fr. 6‐1 and Fr. 6‐2) in the SDGC study, in spite of the presence of substantial signal detected by total Tau ELISA (Figure 9). Therefore, even if highly aggregated Tau species has the binding capability, it may not be so strong binding under physiologically native conditions.

All taken together, AD brain lysate (S1) detected by mAb005 ELISA is concluded to contain at least circa 2000 kD of HMWoTau and more oligomerized tau species, estimated as about 7‐fold longer length on average than HMWoTau. The minimum size of HMWoTau (circa 2000 kD) is in agreement with the description of globular HMWoTau reported as “The approximate molecular mass of the granular tau oligomer averaged 1843.3 ± 112.4 kDa (average ± SD), being corresponding to 40 ± 3 tau molecules.”^43^ We underscore the importance and usefulness to employ SDGC for analysis of highly oligomerized/aggregated tau species with native forms, together with SEC to reach the conclusion.

Regarding the relationship between our purified rhHMWoTau and AD brain lysate‐derived HMWoTau (a minimum size of oligomer Tau), we found three additional evidences besides the same localizations at Fr. 8 (circa 2000 kD) in SEC and at Fr. 3 (circa 2000 kD) in SDGC, at least. These evidences are the rationale to use rhHMWoTau as a calibration standard in mAb005‐ELISAs.

1. Both HMWoTau species were absorbed in a similar manner, by respective IgG‐resin of pan‐tau HT7 (epitope; 159–163 residues) and mAb004 (epitope; N‐terminal of 2N4R tau), besides mAb005's 3D‐form specific epitopes^40^ (Figure 5A,B). These results indicate that the respective antibodies' epitopes are exposed on both molecular surface of rhHMWoTau and AD brain lysate derived‐HMWoTau species.
2. Treatment of both tau species with high concentrations of 2% SDS/2.5 mM DTT reagents eliminated those mAb005‐ELISA signals (Figure 6). These results indicate both tau species are DTT/SDS sensitive molecules and have S–S bonds in the molecular complex. In other words, mAb005 is classified into a conformation‐specific antibody to both HMWoTau species. One difference was that we found a statistically significant increase only in heat‐treated AD brain lysate, while we do not know the reason. The heat treatment might extract more HMWoTau species from AD brain lysate.
3. We could not detect mAb005‐ELISA signals in the supernatant (S2) for both HMWoTau species after high‐speed ultracentrifugation (100 000 *g* for 90 min), in common. This feature was different from that of total Tau and p181Tau species that are collected mainly into S2 (Figure 7).

We cannot ignore that running time on ultracentrifugation is very critical for the fractionation of HMWoTau into S2. The shorter running time (15–60 min) than 90 min was not sufficient to precipitate all HMWoTau species (Figure S5). This may explain the inconsistent results between ours and the relevant report on granular tau oligomer, because they failed to precipitate HMWoTau under 100 000 *g* for 30 min, being insufficient running time for the precipitation based on our data. Besides, the discrepancy could be also due to anti‐tau antibody JM and/or buffers used in the purification and fractionation process.^42^, ^43^

Regarding centrifugation‐based crude purification of exosome, it has been reported that the neuronal exosome contains tau propagation seeds, in which ultracentrifugation at 100 000 *g* for 70 min has been often employed in the purification process.^50^, ^51^ Thus, it needs to be cautiously checked if such fractionated samples may also contain substantial levels of HMWoTau particles (about 30 nm diameter) or not. In fact, we found more than 95% of HMWoTau species fractionated into pellet fraction of P2 under 100 000 *g* even only for 60 min. Therefore, 70 min centrifugation should also precipitate HMWoTau species more than 95%. Based on our findings, we cannot rule out the possibility that the pellet of HMWoTau species could be also included and related to the seed sources on tau propagation (see Figure S7).

In our study, we just simply focused on PBS(−)‐extractable molecules in S1 fraction for analysis of total Tau, p181Tau, and HMWoTau, aiming for quantitation of their native forms, reflecting their original molecular features in physiological/pathological conditions. In fact, we found the slimier ratio of p181Tau to total Tau (approximately 10%) between S1 fraction in human brain lysate (Figures 7G and 10A,B) and CSF.^45^, ^52^ Therefore, we continued to use S1 fraction throughout of our study, in principle.

HMWoTau species, the target molecules of mAb005, showed the most drastic changes depending on the Braak stage at V and VI, compared with total Tau and p181Tau. It is also noteworthy observation that the average level of HMWoTau species in Braak stage I–III was quite low with 8–25 pg/μg total protein (Figure 10C) or 52–60 pg/μg total protein (Figure 10D) in mAb005‐mAb005(Fab’)HRP and mAb004‐mAb005(Fab’)HRP, respectively. These values were detection limits or lower levels of LLOQ. In addition, the highly oligomerized tau oligomer species of NAD's Fr. 4‐2 in SDGC (Figure 9D–F) was only detected by total Tau ELISA, but not by mAb005‐ELISAs. These results indicate that mAb005‐selective HMWoTau species are generated in a disease‐specific manner in the frontal lobe of neocortex, being considered as a final brain region on tau propagation to be accomplished.^35^ Thus, therapeutic use of our disease‐specific mAb005 may show no adverse‐effects in normal subjects who do not have HMWoTau species.

We need to touch on the following noteworthy reports on tau propagation in AD. One article demonstrated that PBS‐extractable AD brain lysate with high‐seeding activity contained higher levels of HMWoTau than those of moderate−/low‐seeding activity in AD subjects using SEC (Superdex 200 10/300 GL; fractionation range from 10 to 600 kD for globular proteins) and a two‐site sandwich ELISA with the same pan‐antibody HT7 as capture and detector,^19^ supporting the link between HMWoTau level and tau‐seeding activity on tau‐propagation. Furthermore, another article showed a Braak‐stage‐dependent increase in tau‐seeding activity in the frontal lobe, while the hippocampus already reached its saturated level at earlier Braak stages, being in line with the tau‐propagation cascade.^53^ Interestingly, immunological absorption of AD brain lysate proteins with mAb005 antibody alleviated tau‐seeding activity in our cell culture studies. This implies the close relationship between HMWoTau species detected by mAb005 and seeding activity (submitted).

We clarified that Thio‐S could also recognize globular particles of rhHMWoTau, as demonstrated by two different technologies of (a) SEC and Thio‐S assay (Figure 1) and (b) dSTORM Nanoimager (Figure 2B). For AD brain lysate‐derived HMWoTau in S1, we further obtained direct evidence that the molecule absorbed by mAb005‐IgG resin showed homogeneous globular particles with a similar average diameter (about 30 nm) labeled with Thio‐S (Submitted). These results indicate the immunologically captured HMWoTau by mAb005 has β‐sheet structures in its oligomer molecule for both rhTau‐derived and AD brain lysate‐derived HMWoTau, in common. Thus, thioflavin‐based assay is impossible to discriminate HMWoTau and its more aggregated tau species such as filamentous tau aggregates, as also suggested in an earlier report.^43^

Because mAb005's target rhHMWoTau was labeled by Thio‐S, we also examined and elucidated that mAb005 has no cross‐reactivity to other proteins that are known to have β‐sheet structure, such as Aβ/α‐synuclein oligomer and fibrils (Figure 4). The results clearly differentiated mAb005 from other reported antibodies such as polyclonal antibodies of A11,^54^ a rabbit monoclonal antibody M204 originated from A11^30^ and a monoclonal antibody GW23B7,^55^ all of which have been reported to have affinities to β‐sheet structures irrespective of protein species.

The successful quantitation of HMWoTau species, total Tau, and p181Tau in tissue‐derived samples seem to lie in the control of non‐specific bindings mainly due to Fc‐binding proteins as well as HAMA (human anti‐mouse antibody) interfering proteins existing in human‐derived samples such as blood, brain, and CSF. Actually, most studies in ELISA or relevant assays do not always use antigen‐specific Fab’ fragment antibody, lacking almost all Fc‐fragment regions of IgG, while HAMA blockers are recently used in most human blood/CSF studies. For example, when mAb005(Fab’)HRP was not used in human lysate studies, non‐specific ELISA signals increased, particularly in one site direct ELISA (data not shown). Therefore, we used a detector antibody of Fab’ fragment directly conjugated with HRP. The conjugation was conducted via SH‐functional moiety of Fab’ fragment to avoid possible inactivation of antigen recognition sites through amine‐ or carboxy‐moieties by cross‐linking reaction. With the efforts above mentioned, we were able to achieve the quantitative measurement of total Tau and p181Tau, besides HMWoTau in tissue‐derived samples.

We could also achieve successful fractionation and quantitation of tau aggregate species by Sucrose‐Density Gradient‐Centrifugation (SDGC),^42^, ^43^ in combination with Tau ELISA. The conditions of 200 000 *g* for 2 h under stepwise‐SDGC (10%–50%, w/v) were quite stringent to reproduce the reported results. For example, under careful layering 10%–50% sucrose solution and collecting each fraction on ice, either change of longer running time than 2 h for centrifugation, higher g‐force than 200 000 *g*, or preparation of sucrose density other unit than w/v (%) shifted HMWoTau localization from Fr. 3 to different fractions, or loading of lower amount of protein resulted in unclear/inconsistent separations (data not shown). Thus, our rhHMWoTau could be a kind of standard reference marker to make sure if SDGC fractionation works successfully or not, by finding the HMWoTau standard at Fr. 3 (30% w/v).

The reason why we did not employ traditional WB analysis to identify mAb005's target protein is the following three reasons. (1) The molecular weight of the target molecules for mAb005 turned out to be circa 2000 kD and above, elucidated by combination analysis with mAb005‐ELISA and SEC/SDGC. Thus, the traditional SDS‐PAGE can't separate such proteins with high MW of circa 2000 kD and above, in principle. (2) WB analysis containing SDS/DTT is not suitable for the analysis of detergent/reduction reagents‐sensitive HMWoTau species, as demonstrated in our ELISA study (Figure 6). (3) When WB analysis was forced to be done, the required brain lysate protein was at least 100 times higher amount than that used in our current ELISA (data not shown). Typically, our highly sensitive ELISAs require 1–30 ng range of brain lysates protein/well (Figure 5C). By contrast, WB analysis requires 1–20 μg range levels of lysate protein/well in general.

In sum, WB analysis will result in the detection of lower affinity of molecules such as disassembled tau bands (data not shown), leading to a kind of misleading conclusion on HMWoTau analysis. To put it the other way around, a highly sensitive ELISA can analyze multiple samples at a time and higher affinity target molecules in native molecular forms.

Lastly, it needs to be addressed on oligomer tau antibodies reported in the past to understand the difference/relevance between mAb005 and other oligomer antibodies. (1) A mouse monoclonal antibody TOC‐1 (IgM) has been identified to bind to high molecular size of oligomers as well as a dimer by WB analysis, and it also preferentially binds to globular oligomers based on morphological analysis. However, there are no MW size data other than WB analysis for the target protein.^56^, ^57^ (2) TOMA (IgG_2_a) has been also reported as a tau oligomer antibody. WB analysis detected the dimer and trimmer tau oligomer species except the monomer. One‐site direct ELISA showed a very marginal but substantial detection of fibrils, implying higher molecular weight of tau species might be also recognized by TOMA.^29^, ^58^ (3) Rabbit polyclonal antibody of T22 (IgG) has been identified to have affinities to LMWoTau species such as dimer/trimmers (110/195 kD) using one‐site direct ELISA and SEC.^15^, ^28^ Traumatic brain injury showed additional higher molecular weight oligomer of 300 kD, besides dimer/trimmers of 75–150 kD.^48^ Thus, T22 seems to have target molecules of more than one species not only LMWoTau species but also the higher molecular weight of oligomers, as also clearly demonstrated by our current study (Figure S7G). (4) A rabbit monoclonal antibody M204 (IgG_1_) has been reported to bind tau oligomers, while it seems to have at least two peaks by analysis with SEC and one‐site ELISA as shown in the report at the position of Fr. 10.5 and Fr. 15.5 mL.^30^ In addition, MA204 has been demonstrated to recognize not only tau oligomers but also other protein species of oligomers from Aβ peptide and prion protein.^59^ Thus, it is considered to be difficult to quantitate one tau oligomer species using MA204. (5) A mouse monoclonal antibody MC1 (IgG_1_) has been identified as a conformation‐specific anti‐tau antibody that decorates globular oligomers as well as longer filamentous tau aggregates by morphological evaluations; however, the detailed characterization of its target molecule species including molecular size has not been disclosed well^26^, ^60^ except dot blot study of fractionated AD brain lysate by SDGC,^43^ where all kinds of tau oligomers species as well as fibrils (from Fr. 2 to Fr. 6) are shown to be MC1‐positive, being different from the recognition pattern by mAb005 on SDGC fractionated samples (Figure 9E,F).

**FIGURE 11:**
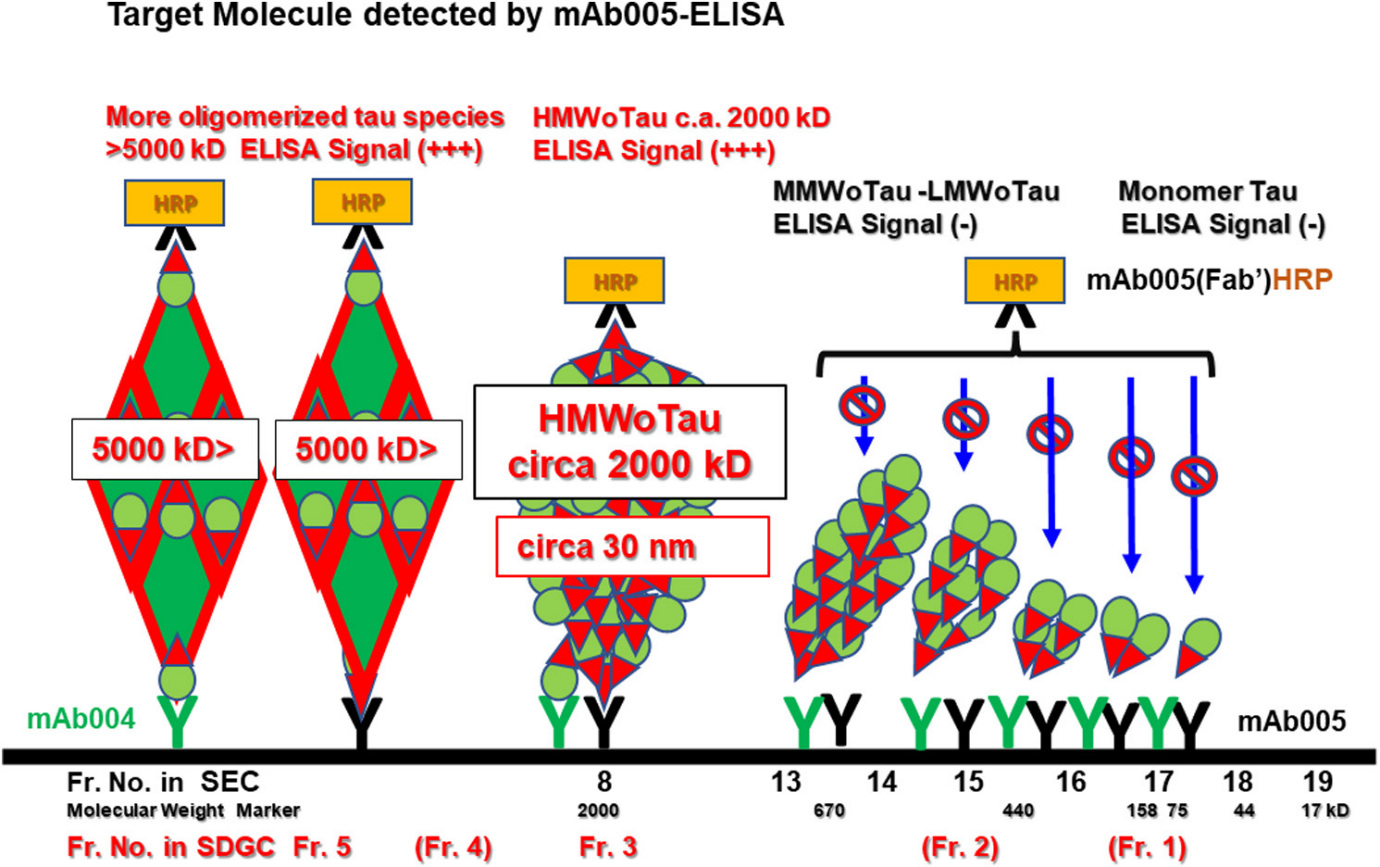
Schematic picture of the target molecule of mAb005‐ELISA. mAb005 detected HMWoTau globular particle with approximately 2000 kD located at Fr. 8 in Size‐Exclusion‐Chromatography (SEC) as well as Fr. 3‐2 in Sucrose‐Density‐Gradient‐Centrifugation (SDGC) and its more oligomerized form above 5000 kD located at Fr. 5‐1 in SDGC (roughly estimated as about 7‐fold longer length on average than that of HMWoTau) in AD specific manner. mAb005‐ELISA did not detect high molecular weight of oligomer tau species of NAD located at Fr. 4‐2 in SDGC, nor the tau species below 2000 kD for both NAD and AD.

In sum, mAb005 is the first and novel antibody that selectively recognizes its target molecule of HMWoTau (circa 2000 kD) as a minimum size of oligomer, together with more oligomerized tau species located at Fr. 5‐1 in SDGC (roughly estimated as about 7‐fold longer length on average than HMWoTau), with no detection of lower tau species below 2000 kD for both AD and NAD as well as no detection of NAD's higher molecular weight of tau species located at Fr. 4‐2 (>5000 kD, between Fr. 3 and Fr. 5‐1 in SDGC) (see Schematic illustration of Figure 11). These mAb005's target molecules of HMWoTau species show up dramatically at Braak stages V and VI in the frontal lobe of AD, indicating the disease‐specific generations. Our highly sensitive and novel mAb005‐ELISA will be useful not only for the further elucidation of etiological roles of HMWoTau species in tau‐associated neurodegenerations, but also for application to its potential diagnostics.

## AUTHOR CONTRIBUTIONS

Conceptualization and design of study: H.F., Authorization and endorsement of study: C.‐Y.T., M.M. and M.‐K.J., Conduct of research and acquisition of samples/data: T.‐H.K. and H.F., Analysis and interpretation of data: H.F., Drafting of article: H.F., Review and revisions of draft: All authors are listed on the title page.

## FUNDING INFORMATION

The research presented in this study was funded by APRINOIA Therapeutics. The Sun City Brain and Body Donation Program has been supported by the National Institute of Neurological Disorders and Stroke (U24 NS072026 National Brain and Tissue Resource for Parkinson's Disease and Related Disorders), the National Institute on Aging (P30 AG19610 and P30 AG072980, Arizona Alzheimer's Disease Center), the Arizona Department of Health Services (contract 211002, Arizona Alzheimer's Research Center), the Arizona Biomedical Research Commission (contracts 4001, 0011, 05‐901 and 1001 to the Arizona Parkinson's Disease Consortium), and the Michael J. Fox Foundation for Parkinson's Research.

## DISCLOSURES

All authors except M.‐K.J. is an employee of APRINOIA Therapeutics Inc., and M.‐K.J. is the founder of this start‐up company.

## Supporting information


Figure S1.

Figure S2.

Figure S3.

Figure S4.

Figure S5.

Figure S6.

Figure S7.

Table S1.

## Data Availability

The data that support the findings of this study are available in the methods and results sections of this article. Other raw data for this study is available from the corresponding author upon request.

## References

[1] Kim CK , Lee YR , Ong L , Gold M , Kalali A , Sarkar J . Alzheimer's disease: key insights from two decades of clinical trial failures. J Alzheimers Dis. 2022;87(1):83‐100. doi:10.3233/JAD-215699 35342092 PMC9198803

[2] Jack CR Jr , Knopman DS , Jagust WJ , et al. Hypothetical model of dynamic biomarkers of the Alzheimer's pathological cascade. Lancet Neurol. 2010;9(1):119‐128. doi:10.1016/S1474-4422(09)70299-6 20083042 PMC2819840

[3] Ingelsson M , Fukumoto H , Newell KL , et al. Early Abeta accumulation and progressive synaptic loss, gliosis, and tangle formation in AD brain. Neurology. 2004;62(6):925‐931. doi:10.1212/01.wnl.0000115115.98960.37 15037694

[4] van Dyck CH , Swanson CJ , Aisen P , et al. Lecanemab in early Alzheimer's disease. N Engl J Med. 2023;388(1):9‐21. doi:10.1056/NEJMoa2212948 36449413

[5] Sims JR , Zimmer JA , Evans CD , et al. Donanemab in early symptomatic Alzheimer disease: the TRAILBLAZER‐ALZ 2 randomized clinical trial. JAMA. 2023;330(6):512‐527. doi:10.1001/jama.2023.13239 37459141 PMC10352931

[6] Schwarz AJ , Yu P , Miller BB , et al. Regional profiles of the candidate tau PET ligand 18F‐AV‐1451 recapitulate key features of Braak histopathological stages. Brain. 2016;139(Pt 5):1539‐1550. doi:10.1093/brain/aww023 26936940

[7] Zhao Q , Liu M , Ha L , Zhou Y , Alzheimer's Disease Neuroimaging Initiative . Quantitative (18)F‐AV1451 brain tau PET imaging in cognitively normal older adults, mild cognitive impairment, and Alzheimer's disease patients. Front Neurol. 2019;10:486. doi:10.3389/fneur.2019.00486 31156534 PMC6530456

[8] Timmers T , Ossenkoppele R , Wolters EE , et al. Associations between quantitative [(18)F]flortaucipir tau PET and atrophy across the Alzheimer's disease spectrum. Alzheimers Res Ther. 2019;11(1):60. doi:10.1186/s13195-019-0510-3 31272512 PMC6610969

[9] Tanner JA , Iaccarino L , Edwards L , et al. Amyloid, tau and metabolic PET correlates of cognition in early and late‐onset Alzheimer's disease. Brain. 2022;145:4489‐4505. doi:10.1093/brain/awac229 35762829 PMC10200306

[10] Devous MD Sr , Fleisher AS , Pontecorvo MJ , et al. Relationships between cognition and neuropathological tau in Alzheimer's disease assessed by 18F Flortaucipir PET. J Alzheimers Dis. 2021;80(3):1091‐1104. doi:10.3233/JAD-200808 33682705

[11] Biel D , Brendel M , Rubinski A , et al. Tau‐PET and in vivo Braak‐staging as prognostic markers of future cognitive decline in cognitively normal to demented individuals. Alzheimers Res Ther. 2021;13(1):137. doi:10.1186/s13195-021-00880-x 34384484 PMC8361801

[12] Fukumoto H , Asami‐Odaka A , Suzuki N , Shimada H , Ihara Y , Iwatsubo T . Amyloid beta protein deposition in normal aging has the same characteristics as that in Alzheimer's disease. Predominance of a beta 42(43) and association of a beta 40 with cored plaques. Am J Pathol. 1996;148(1):259‐265. 8546214 PMC1861616

[13] Kundel F , Tosatto L , Whiten DR , Wirthensohn DC , Horrocks MH , Klenerman D . Shedding light on aberrant interactions—a review of modern tools for studying protein aggregates. FEBS J. 2018;285(19):3604‐3630. doi:10.1111/febs.14409 29453901

[14] DeVos SL , Corjuc BT , Oakley DH , et al. Synaptic tau seeding precedes tau pathology in human Alzheimer's disease brain. Front Neurosci. 2018;12:267. doi:10.3389/fnins.2018.00267 29740275 PMC5928393

[15] Lasagna‐Reeves CA , Castillo‐Carranza DL , Sengupta U , et al. Alzheimer brain‐derived tau oligomers propagate pathology from endogenous tau. Sci Rep. 2012;2:700. doi:10.1038/srep00700 23050084 PMC3463004

[16] Stopschinski BE , Del Tredici K , Estill‐Terpack SJ , et al. Anatomic survey of seeding in Alzheimer's disease brains reveals unexpected patterns. Acta Neuropathol Commun. 2021;9(1):164. doi:10.1186/s40478-021-01255-x 34635189 PMC8507321

[17] Martinez P , Patel H , You Y , et al. Bassoon contributes to tau‐seed propagation and neurotoxicity. Nat Neurosci. 2022;25(12):1597‐1607. doi:10.1038/s41593-022-01191-6 36344699 PMC9708566

[18] Takeda S , Wegmann S , Cho H , et al. Neuronal uptake and propagation of a rare phosphorylated high‐molecular‐weight tau derived from Alzheimer's disease brain. Nat Commun. 2015;6:8490. doi:10.1038/ncomms9490 26458742 PMC4608380

[19] Dujardin S , Commins C , Lathuiliere A , et al. Tau molecular diversity contributes to clinical heterogeneity in Alzheimer's disease. Nat Med. 2020;26(8):1256‐1263. doi:10.1038/s41591-020-0938-9 32572268 PMC7603860

[20] Wells C , Brennan S , Keon M , Ooi L . The role of amyloid oligomers in neurodegenerative pathologies. Int J Biol Macromol. 2021;181:582‐604. doi:10.1016/j.ijbiomac.2021.03.113 33766600

[21] Trease AJ , George JW , Roland NJ , et al. Hyperphosphorylated human tau accumulates at the synapse, localizing on synaptic mitochondrial outer membranes and disrupting respiration in a mouse model of tauopathy. Front Mol Neurosci. 2022;15:852368. doi:10.3389/fnmol.2022.852368 35359570 PMC8960727

[22] Shafiei SS , Guerrero‐Muñoz MJ , Castillo‐Carranza DL . Tau oligomers: cytotoxicity, propagation, and mitochondrial damage. Front Aging Neurosci. 2017;9:83. doi:10.3389/fnagi.2017.00083 28420982 PMC5378766

[23] Niewiadomska G , Niewiadomski W , Steczkowska M , Gasiorowska A . Tau oligomers neurotoxicity. Life (Basel). 2021;11(1):28. doi:10.3390/life11010028 33418848 PMC7824853

[24] Stern AM , Selkoe DJ . Soluble oligomers or insoluble fibrils? Scientific commentary on “tau seeding and spreading in vivo is supported by both AD‐derived fibrillar and oligomeric tau”. Acta Neuropathol. 2023;146:861‐862. doi:10.1007/s00401-023-02633-6 37733037

[25] Mate de Gerando A , Quittot N , Frosch MP , Hyman BT . Reply: soluble oligomers or insoluble fibrils? Acta Neuropathol. 2023;146:863‐866. doi:10.1007/s00401-023-02634-5 37733036 PMC10628010

[26] Charles L , Weaverb ME , Kressa Y , Daviesa P . Conformational change as one of the earliest alterations of tau in Alzheimer's disease. Neurobiol Aging. 2000;21:719‐727. 11016541 10.1016/s0197-4580(00)00157-3

[27] Ward SM , Himmelstein DS , Lancia JK , Fu Y , Patterson KR , Binder LI . TOC1: characterization of a selective oligomeric tau antibody. J Alzheimers Dis. 2013;37(3):593‐602. doi:10.3233/JAD-131235 23979027 PMC4791958

[28] Lasagna‐Reeves CA , Castillo‐Carranza DL , Sengupta U , et al. Identification of oligomers at early stages of tau aggregation in Alzheimer's disease. FASEB J. 2012;26(5):1946‐1959. doi:10.1096/fj.11-199851 22253473 PMC4046102

[29] Castillo‐Carranza DL , Gerson JE , Sengupta U , Guerrero‐Munoz MJ , Lasagna‐Reeves CA , Kayed R . Specific targeting of tau oligomers in Htau mice prevents cognitive impairment and tau toxicity following injection with brain‐derived tau oligomeric seeds. J Alzheimers Dis. 2014;40(suppl. 1):S97‐S111. doi:10.3233/JAD-132477 24603946

[30] Abskharon R , Seidler PM , Sawaya MR , et al. Crystal structure of a conformational antibody that binds tau oligomers and inhibits pathological seeding by extracts from donors with Alzheimer's disease. J Biol Chem. 2020;295(31):10662‐10676. doi:10.1074/jbc.RA120.013638 32493775 PMC7397112

[31] Santacruz K , Lewis J , Spires T , et al. Tau suppression in a neurodegenerative mouse model improves memory function. Science. 2005;309(5733):476‐481. doi:10.1126/science.1113694 16020737 PMC1574647

[32] Sahara N , DeTure M , Ren Y , et al. Characteristics of TBS‐extractable hyperphosphorylated tau species: aggregation intermediates in rTg4510 mouse brain. J Alzheimers Dis. 2013;33(1):249‐263. doi:10.3233/JAD-2012-121093 22941973 PMC3514650

[33] Schroeder S , Joly‐Amado A , Soliman A , et al. Oligomeric tau‐targeted immunotherapy in Tg4510 mice. Alzheimers Res Ther. 2017;9(1):46. doi:10.1186/s13195-017-0274-6 28655349 PMC5488475

[34] Wegmann S , Nicholls S , Takeda S , Fan Z , Hyman BT . Formation, release, and internalization of stable tau oligomers in cells. J Neurochem. 2016;139(6):1163‐1174. doi:10.1111/jnc.13866 27731899 PMC5283951

[35] Xu X , Ruan W , Liu F , et al. (18)F‐APN‐1607 tau positron emission tomography imaging for evaluating disease progression in Alzheimer's disease. Front Aging Neurosci. 2021;13:789054. doi:10.3389/fnagi.2021.789054 35221982 PMC8868571

[36] Tagai K , Ono M , Kubota M , et al. High‐contrast in vivo imaging of tau pathologies in Alzheimer's and non‐Alzheimer's disease tauopathies. Neuron. 2021;109(1):42‐58.e8. doi:10.1016/j.neuron.2020.09.042 33125873

[37] Miyamoto M , Okuyama C , Kagawa S , et al. Radiation dosimetry and pharmacokinetics of the tau PET tracer florzolotau (18F) in healthy Japanese subjects. Ann Nucl Med. 2023;37(5):300‐309. doi:10.1007/s12149-023-01828-x 36890399 PMC10129982

[38] Endo H , Tagai K , Ono M , et al. A machine learning–based approach to discrimination of tauopathies using [18F] PM‐PBB3 PET images. Mov Disord. 2022;37:2236‐2246. doi:10.1002/mds.29173 36054492 PMC9805085

[39] Quinn JP , Corbett NJ , Kellett KAB , Hooper NM . Tau proteolysis in the pathogenesis of tauopathies: neurotoxic fragments and novel biomarkers. J Alzheimers Dis. 2018;63(1):13‐33. doi:10.3233/JAD-170959 29630551 PMC5900574

[40] Zheng H , Sun H , Cai Q , Tai HC . The enigma of tau protein aggregation: mechanistic insights and future challenges. Int J Mol Sci. 2024;25(9):4969. doi:10.3390/ijms25094969 38732197 PMC11084794

[41] Devred F , Barbier P , Douillard S , Monasterio O , Andreu JM , Peyrot V . Tau induces ring and microtubule formation from αβ‐tubulin dimers under nonassembly conditions. Biochemistry. 2004;43(32):10520‐10531. doi:10.1021/bi0493160 15301550

[42] Maeda S , Sahara N , Saito Y , Murayama S , Ikai A , Takashima A . Increased levels of granular tau oligomers: an early sign of brain aging and Alzheimer's disease. Neurosci Res. 2006;54(3):197‐201. doi:10.1016/j.neures.2005.11.009 16406150

[43] Maeda S , Sahara N , Saito Y , et al. Granular tau oligomers as intermediates of tau filaments. Biochemistry. 2007;46(12):3856‐3861. doi:10.1021/bi061359o 17338548

[44] Wang P , Ye Y . Filamentous recombinant human tau activates primary astrocytes via an integrin receptor complex. Nat Commun. 2021;12(1):95. doi:10.1038/s41467-020-20322-w 33398028 PMC7782792

[45] Kawarabayashi T , Nakamura T , Miyashita K , Fukamachi I , Seino Y , Shoji M . Novel ELISAs to measure total and phosphorylated tau in cerebrospinal fluid. Neurosci Lett. 2020;722:134826. doi:10.1016/j.neulet.2020.134826 32045623

[46] Fukumoto H , Tokuda T , Kasai T , et al. High‐molecular‐weight beta‐amyloid oligomers are elevated in cerebrospinal fluid of Alzheimer patients. FASEB J. 2010;24(8):2716‐2726. doi:10.1096/fj.09-150359 20339023

[47] Beach TG , Adler CH , Sue LI , et al. Arizona study of aging and neurodegenerative disorders and brain and body donation program. Neuropathology. 2015;35(4):354‐389. doi:10.1111/neup.12189 25619230 PMC4593391

[48] Hawkins BE , Krishnamurthy S , Castillo‐Carranza DL , et al. Rapid accumulation of endogenous tau oligomers in a rat model of traumatic brain injury: possible link between traumatic brain injury and sporadic tauopathies. J Biol Chem. 2013;288(23):17042‐17050. doi:10.1074/jbc.M113.472746 23632019 PMC3675635

[49] Thacker D , Barghouth M , Bless M , Zhang E , Linse S . Direct observation of secondary nucleation along the fibril surface of the amyloid β 42 peptide. Proc Natl Acad Sci USA. 2023;120(25):e2220664120. doi:10.1073/pnas.2220664120 37307445 PMC10288637

[50] Wang Y , Balaji V , Kaniyappan S , et al. The release and trans‐synaptic transmission of tau via exosomes. Mol Neurodegener. 2017;12(1):1‐25. doi:10.1186/s13024-016-0143-y 28086931 PMC5237256

[51] Ruan Z , Delpech JC , Venkatesan Kalavai S , et al. P2RX7 inhibitor suppresses exosome secretion and disease phenotype in P301S tau transgenic mice. Mol Neurodegener. 2020;15(1):47. doi:10.1186/s13024-020-00396-2 32811520 PMC7436984

[52] Lehmann S , Dumurgier J , Ayrignac X , et al. Cerebrospinal fluid A beta 1‐40 peptides increase in Alzheimer’s disease and are highly correlated with phospho‐tau in control individuals. Alzheimers Res Ther. 2020;12(1):123. doi:10.1186/s13195-020-00696-1 PMC7532565 33008460

[53] Furman JL , Vaquer‐Alicea J , White CL 3rd , Cairns NJ , Nelson PT , Diamond MI . Widespread tau seeding activity at early Braak stages. Acta Neuropathol. 2017;133(1):91‐100. doi:10.1007/s00401-016-1644-z 27878366 PMC5833300

[54] Kayed R , Head E , Sarsoza F , et al. Fibril specific, conformation dependent antibodies recognize a generic epitope common to amyloid fibrils and fibrillar oligomers that is absent in prefibrillar oligomers. Mol Neurodegener. 2007;2:18. doi:10.1186/1750-1326-2-18 17897471 PMC2100048

[55] Goni F , Marta‐Ariza M , Herline K , et al. Anti‐beta‐sheet conformation monoclonal antibody reduces tau and Abeta oligomer pathology in an Alzheimer's disease model. Alzheimers Res Ther. 2018;10(1):1‐16. doi:10.1186/s13195-018-0337-3 29378642 PMC5789573

[56] Patterson KR , Remmers C , Fu Y , et al. Characterization of prefibrillar tau oligomers in vitro and in Alzheimer disease. J Biol Chem. 2011;286(26):23063‐23076. doi:10.1074/jbc.M111.237974 21550980 PMC3123074

[57] Ward SM , Himmelstein DS , Ren Y , et al. TOC1: a valuable tool in assessing disease progression in the rTg4510 mouse model of tauopathy. Neurobiol Dis. 2014;67:37‐48. doi:10.1016/j.nbd.2014.03.002 24631720 PMC4055868

[58] Castillo‐Carranza DL , Sengupta U , Guerrero‐Munoz MJ , et al. Passive immunization with tau oligomer monoclonal antibody reverses tauopathy phenotypes without affecting hyperphosphorylated neurofibrillary tangles. J Neurosci. 2014;34(12):4260‐4272. doi:10.1523/JNEUROSCI.3192-13.2014 24647946 PMC6608097

[59] Arai H , Glabe C , Luecke H . Crystal structure of a conformationdependent rabbit IgG Fab specific for amyloid prefibrillar oligomers. Biochim Biophys Acta. 2012;1820(12):1908‐1914. doi:10.1016/j.bbagen.2012.08.016 22940003 PMC3973424

[60] Rissman RA , Staup MA , Lee AR , et al. Corticotropin‐releasing factor receptor‐dependent effects of repeated stress on tau phosphorylation, solubility, and aggregation. Proc Natl Acad Sci USA. 2012;109(16):6277‐6282. doi:10.1073/pnas.1203140109 22451915 PMC3341026

