# Supplementary material for: High‐molecular‐weight oligomer tau (HMWoTau) species are dramatically increased in Braak‐stage dependent manner in the frontal lobe of human brains, demonstrated by a novel oligomer Tau ELISA with a mouse monoclonal antibody (APNmAb005)
Source: FASEB J. 2024 Nov 20;38(22):e70160. doi: 10.1096/fj.202401704R (PMC11578280; doi:10.1096/fj.202401704R)
Supplement: Supplementary file 1 — Figure S1. [file FSB2-38-e70160-s008.pdf]

Supplemental Figure 1

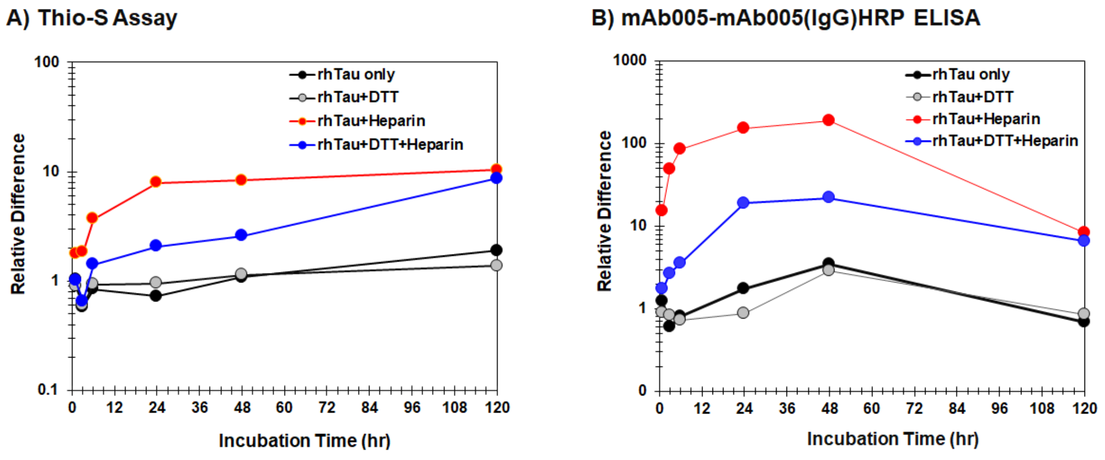

Supplemental Figure 1. Time-dependent generation of Thio-S positive tau aggregate and mAb005-mAb005(IgG)HRP ELISA-positive tau oligomer in four groups of "rhTau only", "rhTau+Heparin", "rhTau+DTT", and "rhTau+Heparin +DTT". rhTau (4 mg/mL) was mixed with vehicle, DTT (2 mM), Heparin (336 μg/mL), or both DTT and Heparin. Then, the mixture was incubated at 37°C without shaking. At each indicated time point, samples were collected and stored at freezer until assay. All of the samples were subjected to **A)** Thio-S assay and **B)** mAb005-mAb005(IgG)HRP ELISA. Each of (A) Thio-S fluorescence intensity and (B) oligomer Tau level was compared with those of no incubated rhTau and expressed as relative difference with a single determination or mean of duplicate as a pilot study, respectively.
