# Supplementary material for: High‐molecular‐weight oligomer tau (HMWoTau) species are dramatically increased in Braak‐stage dependent manner in the frontal lobe of human brains, demonstrated by a novel oligomer Tau ELISA with a mouse monoclonal antibody (APNmAb005)
Source: FASEB J. 2024 Nov 20;38(22):e70160. doi: 10.1096/fj.202401704R (PMC11578280; doi:10.1096/fj.202401704R)
Supplement: Supplementary file 2 — Figure S2. [file FSB2-38-e70160-s004.pdf]

Supplemental Figure 2

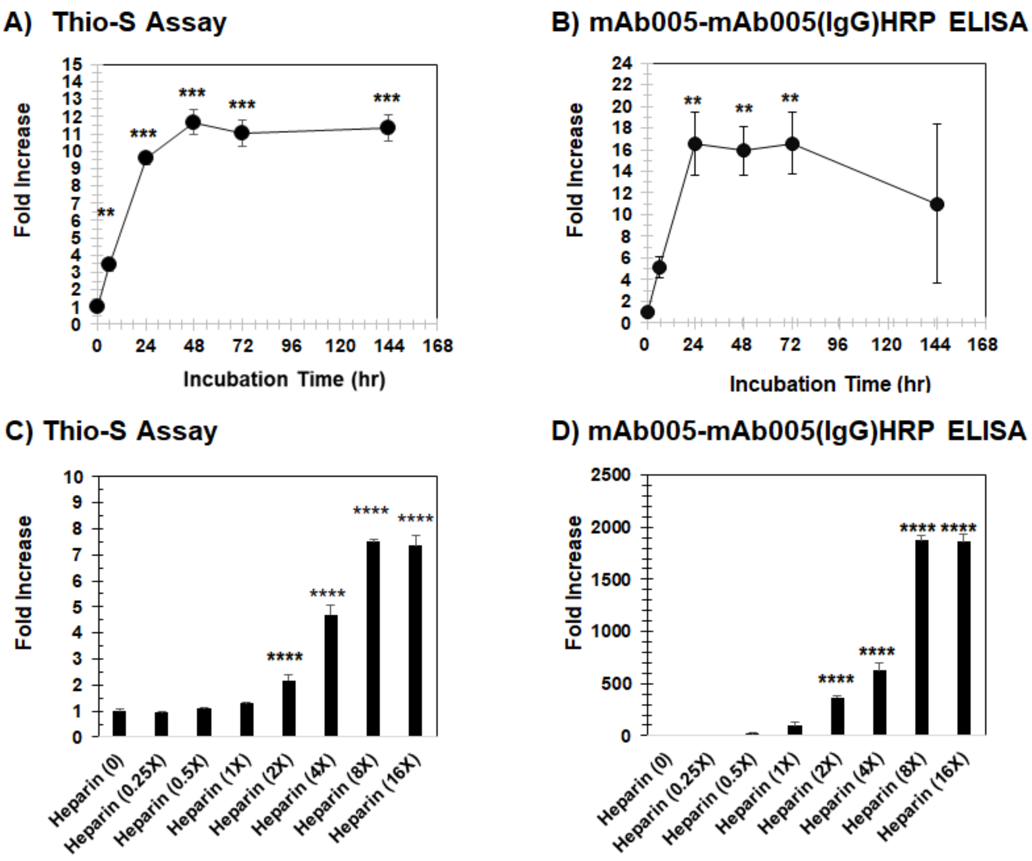

Supplemental Figure 2. Time-dependent and heparin-dependent generation of Thio-S positive aggregates and mAb005-mAb005(IgG)HRP-positive Tau oligomers. For time-course study, rhTau (2N4R) (4 mg/mL) and heparin (400 µg/mL) were incubated for 0.1, 6, 24, 48, 72 and 144 h. Each of the reacted mixture was subjected to **(A)** Thio-S assay or to **(B)** mAb005-mAb005(IgG)HRP ELISA. The 0.1 h incubation was set as a control. For heparin-dependency study, rhTau (2N4R) (4 mg/mL) and heparin (0-640 µg/mL) were incubated for 24 h and each of the reacted mixture was subjected to **(C)** Thio-S assay and **(D)** mAb005-mAb005(IgG)HRP ELISA. No heparin at 24 h incubation was set as a control. Values are expressed as fold increase to control (means±SD of 3 determinations). 1xHepain= 40 µg/mL. *P<0.05,**P<0.01,****<0.001, ****P<0.0001 vs. control (Dunnett’s test).
