# Supplementary material for: High‐molecular‐weight oligomer tau (HMWoTau) species are dramatically increased in Braak‐stage dependent manner in the frontal lobe of human brains, demonstrated by a novel oligomer Tau ELISA with a mouse monoclonal antibody (APNmAb005)
Source: FASEB J. 2024 Nov 20;38(22):e70160. doi: 10.1096/fj.202401704R (PMC11578280; doi:10.1096/fj.202401704R)
Supplement: Supplementary file 3 — Figure S3. [file FSB2-38-e70160-s007.pdf]

Supplemental Figure 3

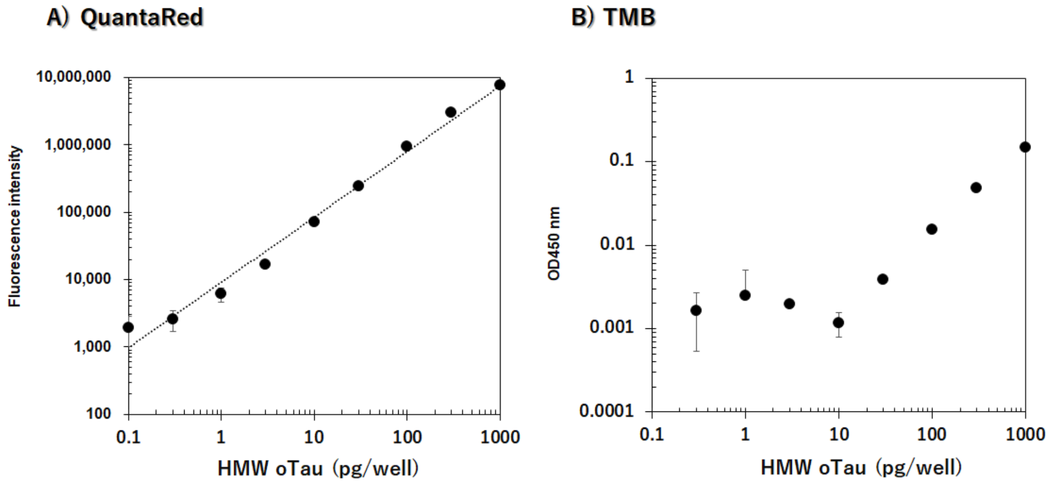

Supplemental Figure 3. Improved detection limit by fluorescent substrate compared with TMB colorimetric substrate in mAb005-mAb005(Fab')HRP ELISA. rhHMWoTau (0.1-1000 pg/well) was subjected to mAb005-mAb005(Fab')HRP ELISA and the enzymatically reacted product was read using **(A)** QuantaRed fluorescent substrate ($\lambda_{Ex}/\lambda_{Em}$=570/585 nm) or **(B)** colorimetric substrate TMB (OD450 nm) after stopping the reaction with 1N phosphoric acid. Values are expressed as means±SD (n=3 determinations) after adjusted with background subtraction.
