# Supplementary material for: High‐molecular‐weight oligomer tau (HMWoTau) species are dramatically increased in Braak‐stage dependent manner in the frontal lobe of human brains, demonstrated by a novel oligomer Tau ELISA with a mouse monoclonal antibody (APNmAb005)
Source: FASEB J. 2024 Nov 20;38(22):e70160. doi: 10.1096/fj.202401704R (PMC11578280; doi:10.1096/fj.202401704R)
Supplement: Supplementary file 4 — Figure S4. [file FSB2-38-e70160-s006.pdf]

Supplemental Figure 4

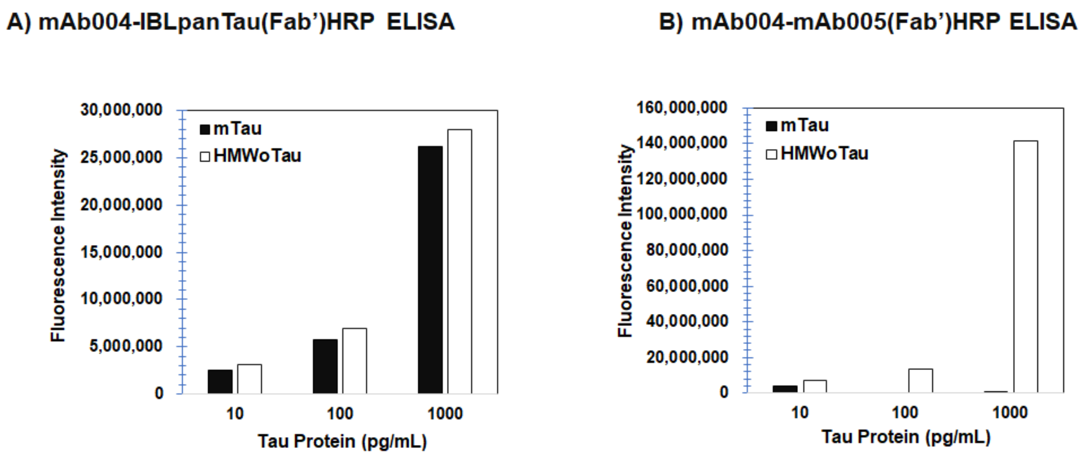

Supplemental Figure 4. Specific detection of HMWoTau in a two-sites sandwich ELISA of mAb004-Ab005(Fab')HRP. Various concentrations of recombinant human 2N4R Tau-derived HMWoTau (HMWoTau) or recombinant human monomer 2N4R Tau (mTau) (IBL, total Tau-kit standard) were subjected to total Tau ELISA of **A)** mAb004-IBLpanTau(Fab')HRP and mAb005-ELISA of **B)** mAb004-mAb005(Fab')HRP. Values are expressed as means of two determinations after adjusted with background subtraction.
