# Supplementary material for: High‐molecular‐weight oligomer tau (HMWoTau) species are dramatically increased in Braak‐stage dependent manner in the frontal lobe of human brains, demonstrated by a novel oligomer Tau ELISA with a mouse monoclonal antibody (APNmAb005)
Source: FASEB J. 2024 Nov 20;38(22):e70160. doi: 10.1096/fj.202401704R (PMC11578280; doi:10.1096/fj.202401704R)
Supplement: Supplementary file 5 — Figure S5. [file FSB2-38-e70160-s002.pdf]

Supplemental Figure 5

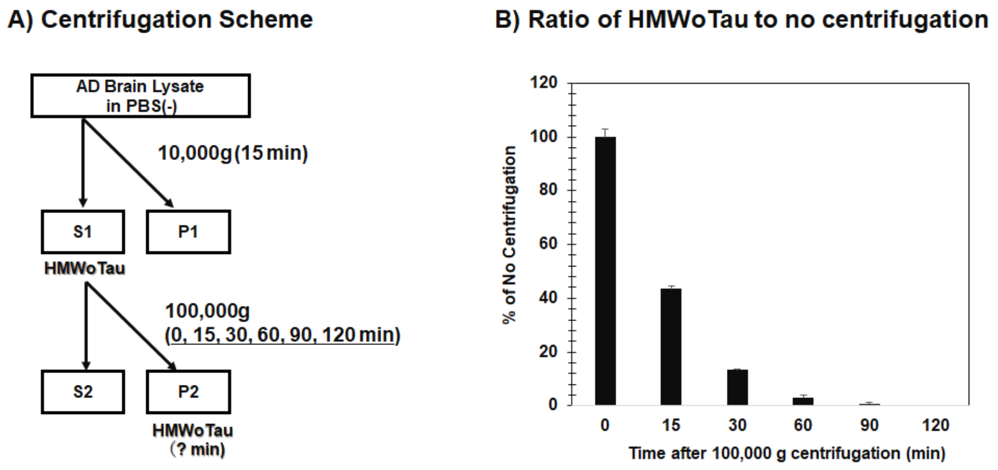

Supplemental Figure 5. Effects of ultracentrifugation running-time on detection of HMWoTau species in supernatant of AD bran lysate after ultracentrifugation.

A) Centrifugation of Scheme; AD brain lysate mixture (S1) was further centrifuged at 100,000g for each indicated time. Each supernatant (S2) was subjected to a two-site sandwich ELISA of Ab005-mAb005(Fab')HRP. **B) Ratio of HMWoTau to no centrifugation**. Values are expressed as means±SD (N=3 determinations) of % of no centrifugation. Complete elimination of HMWoTau species from S2 was observed at 90 min and above.
