# Supplementary material for: High‐molecular‐weight oligomer tau (HMWoTau) species are dramatically increased in Braak‐stage dependent manner in the frontal lobe of human brains, demonstrated by a novel oligomer Tau ELISA with a mouse monoclonal antibody (APNmAb005)
Source: FASEB J. 2024 Nov 20;38(22):e70160. doi: 10.1096/fj.202401704R (PMC11578280; doi:10.1096/fj.202401704R)
Supplement: Supplementary file 6 — Figure S6. [file FSB2-38-e70160-s001.pdf]

Supplemental Figure 6

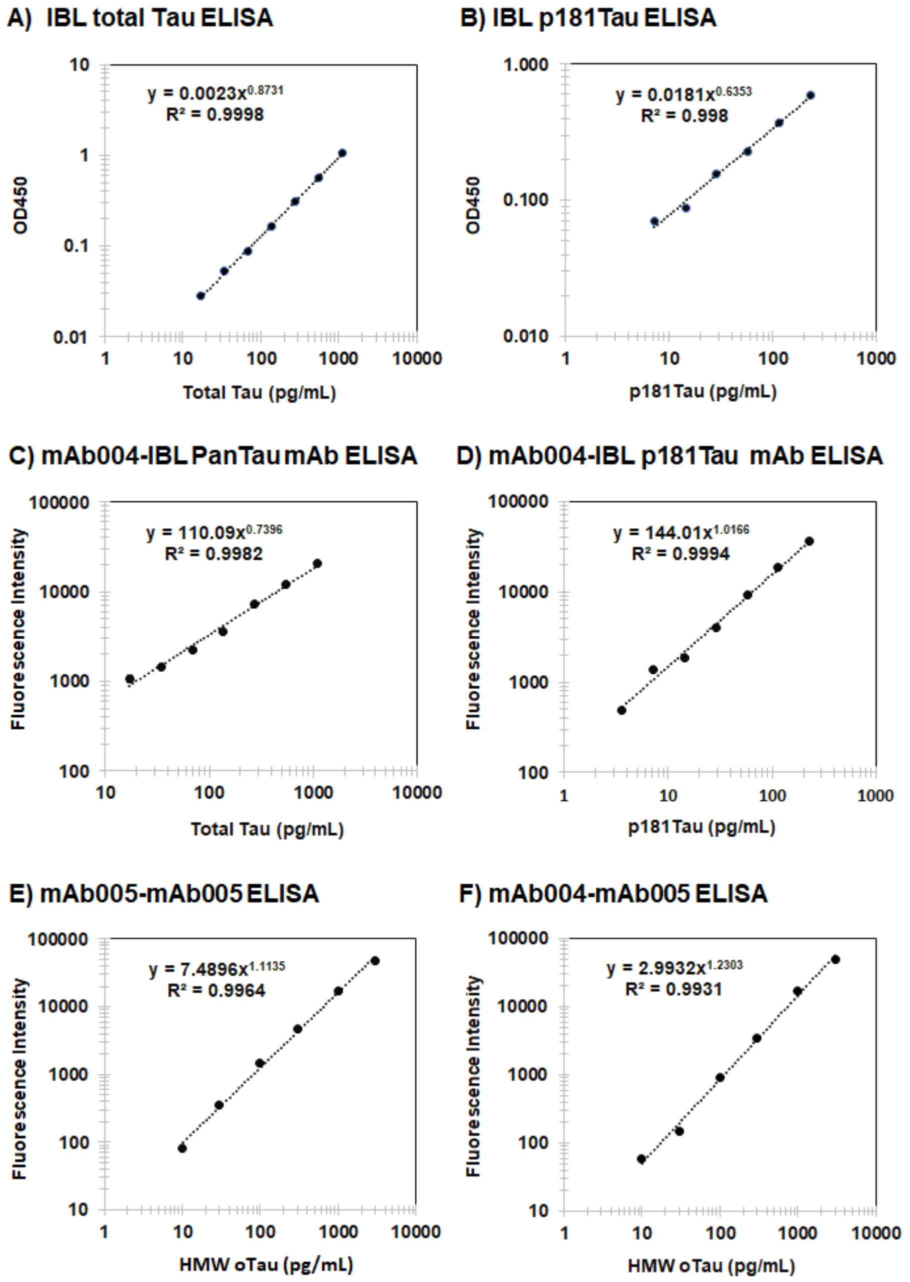

Supplemental Figure 6. Standard Curve for total Tau, p181Tau and HMWoTau. Each standard protein was subjected to respective two site sandwich ELISAs of **A)** IBL total Tau kit, **B)** IBL p181 Tau kit, **C)** mAb004 -IBL panTau(Fab')HRP, **D)** mAb004-IBL p181Tau(Fab')HRP, **E)** mAb005-mAb005(Fab')HRP, or **F)** mAb004-mAb005(Fab')HRP. Values are expressed as means of two determinations after adjusted with background subtraction.
