# Supplementary material for: High‐molecular‐weight oligomer tau (HMWoTau) species are dramatically increased in Braak‐stage dependent manner in the frontal lobe of human brains, demonstrated by a novel oligomer Tau ELISA with a mouse monoclonal antibody (APNmAb005)
Source: FASEB J. 2024 Nov 20;38(22):e70160. doi: 10.1096/fj.202401704R (PMC11578280; doi:10.1096/fj.202401704R)
Supplement: Supplementary file 7 — Figure S7. [file FSB2-38-e70160-s003.pdf]

Supplemental Figure 7

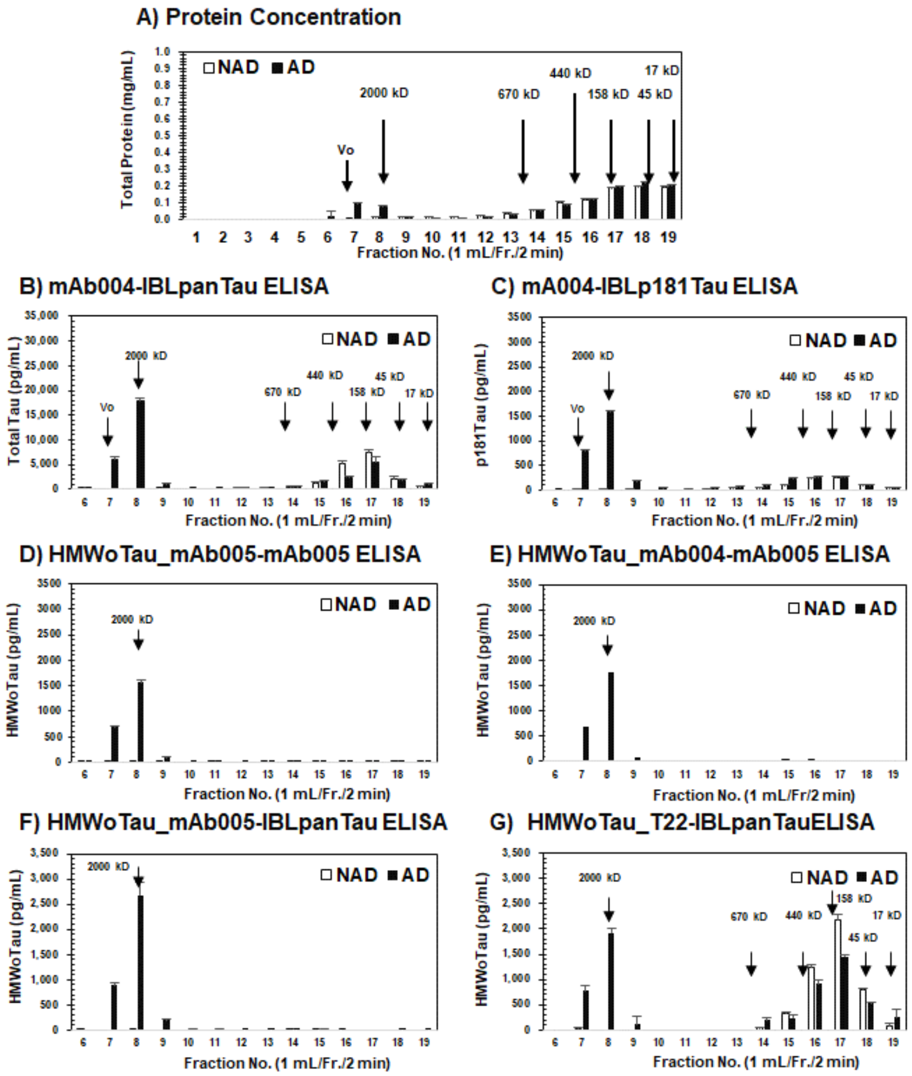

Supplemental Figure 7. Selective detection of HMWoTau (circa 2000 kD) by mAb005-ELISAs under the presence of multiple tau species detected by ELISAs of oligomer antibody T22, total Tau, and p181Tau. Brain lysates mixtures were fractionated by SEC on new age-matched representatives of NAD (n=6) & AD (n=6), and **(A)** each fractionated protein concentration was determined with OD280 using BSA as a standard. Then, each sample was subjected to respective two-site sandwich ELISAs of **(B)** total Tau, mAb004-IBLpanTau(Fab')HRP, **(C)** p181Tau, mAb004-IBLp181Tau(Fab')HRP, **(D)** mAb005-mAb005(Fab')HRP, **(E)** mAb004-mAb005(Fab')HRP, **(F)** mAb005-IBLpanTau(Fab')HRP or **(G)**T22-IBLpanTau(Fab')HRP. Values are means±SD (N=3 determinations). Vo; Void Volume.
