# Supplementary material for: High‐molecular‐weight oligomer tau (HMWoTau) species are dramatically increased in Braak‐stage dependent manner in the frontal lobe of human brains, demonstrated by a novel oligomer Tau ELISA with a mouse monoclonal antibody (APNmAb005)
Source: FASEB J. 2024 Nov 20;38(22):e70160. doi: 10.1096/fj.202401704R (PMC11578280; doi:10.1096/fj.202401704R)
Supplement: Supplementary file 8 — Table S1. [file FSB2-38-e70160-s005.docx]

**Supplemental Table**

**Supplemental Table 1.** **Demographic Data of representatives on AD and NAD subjects (N=6)**

| **No.** | **Braak Stage** | **Age** | **Gender** | **PMI  (h)** | **Apo E Genotype** |
| --- | --- | --- | --- | --- | --- |
| **2** | **I** | **75** | **M** | **3.33** | **3/3** |
| **4** | **I** | **77** | **F** | **3.25** | **2/3** |
| **5** | **I** | **78** | **F** | **1.25** | **3/3** |
| **8** | **I** | **63** | **M** | **4.16** | **3/3** |
| **9** | **I** | **82** | **M** | **1.66** | **3/3** |
| **10** | **I** | **81** | **F** | **2.75** | **2/3** |
| **31** | **V** | **87** | **F** | **3.00** | **3/3** |
| **34** | **V** | **74** | **F** | **2.00** | **3/4** |
| **36** | **VI** | **64** | **F** | **3.16** | **3/4** |
| **37** | **VI** | **75** | **M** | **2.25** | **3/3** |
| **39** | **VI** | **76** | **F** | **2.00** | **3/3** |
| **40** | **VI** | **89** | **F** | **4.00** | **3/3** |

M:Male; F, Female; PMI, Postmortem Interval
